# Cell cycle-resolved chromatin proteomics reveals the extent of mitotic preservation of the genomic regulatory landscape

**DOI:** 10.1038/s41467-018-06007-5

**Published:** 2018-10-02

**Authors:** Paul Adrian Ginno, Lukas Burger, Jan Seebacher, Vytautas Iesmantavicius, Dirk Schübeler

**Affiliations:** 1 Friedrich Miescher Institute for Biomedical Research, Basel, Switzerland; 2 Swiss Institute of Bioinformatics, Basel, Switzerland; 3 Faculty of Science, University of Basel, Basel, Switzerland

## Abstract

Regulation of transcription, replication, and cell division relies on differential protein binding to DNA and chromatin, yet it is unclear which regulatory components remain bound to compacted mitotic chromosomes. By utilizing the buoyant density of DNA–protein complexes after cross-linking, we here develop a mass spectrometry-based approach to quantify the chromatin-associated proteome at separate stages of the cell cycle. While epigenetic modifiers that promote transcription are lost from mitotic chromatin, repressive modifiers generally remain associated. Furthermore, while proteins involved in transcriptional elongation are evicted, most identified transcription factors are retained on mitotic chromatin to varying degrees, including core promoter binding proteins. This predicts conservation of the regulatory landscape on mitotic chromosomes, which we confirm by genome-wide measurements of chromatin accessibility. In summary, this work establishes an approach to study chromatin, provides a comprehensive catalog of chromatin changes during the cell cycle, and reveals the degree to which the genomic regulatory landscape is maintained through mitosis.

## Abstract (web summary)

Mitosis poses a challenge for transcriptional programs, as it is thought that several proteins lose binding on condensed chromosomes. Here, the authors analyze the chromatin-bound proteome through the cell cycle, revealing retention of most transcription factors and preservation of the regulatory landscape.

## Introduction

Differential recruitment of proteins to chromatin is fundamental to any DNA-templated event in eukaryotes, and thus chromatin composition directly reflects cellular state and identity. One such example of changing cellular states is progression through the cell cycle, a process accompanied by dynamic changes on chromatin, including transcription, DNA replication, DNA damage repair, and chromosomal condensation for segregation into daughter cells^1–4^. More specifically, the condensation of chromosomes during mitosis represents a dramatic change in chromatin organization and is proposed to coincide with widespread eviction of chromatin-associated proteins^5^. However, selected factors are thought to be retained and index particular genomic regions to facilitate transcriptional activation upon mitotic exit. This concept is potentially relevant for maintenance of cellular identity and is termed mitotic bookmarking^6^.

Mediators of such bookmarking include transcription factor (TF) binding, epigenetic marks and local chromatin structure^7,8^. Loss of TFs from mitotic chromatin has been observed in several studies^9–13^, however, recent live cell imaging has called the generality of this model into question^14,15^. Additionally, there are several conflicting reports regarding the retention of chromatin proteins throughout mitosis^8^. While efforts have been made to catalogue the constituents of mitotic chromatin^16^, we still lack a comprehensive analysis contrasting bulk changes of chromatin proteins between M- and G1-phase and relating it to the persistence of regulatory regions.

There are inherent difficulties to biochemically enrich chromatin for quantitative analysis, likely due to its highly charged nature^16^, a property that creates uncertainty in defining chromatin functions for a given protein^17^. Despite this, considerable information regarding the genomic locations of proteins has been garnered using ChIP-seq^18^, a technique that utilizes formaldehyde cross-linking to preserve chromatin protein interactions. While widely used, including in large scale epigenomic efforts^19^, it is restricted to known targets and one factor per experiment. Here, we analyze the protein content of formaldehyde cross-linked chromatin using tandem mass tag (TMT)^20^ multiplexing and high-resolution mass spectrometry (MS). We have termed the method density-based enrichment for mass spectrometry analysis of chromatin (DEMAC), and utilize it to quantify changes in the chromatin-bound proteome (chromatome) across G1-, S-, and M-phase of the human cell cycle. In addition to providing a rich dataset of chromatin composition during the cell cycle, our results reveal pathway-specific retention of chromatin modifiers on mitotic chromosomes, including a widespread retention of TFs.

## Results

### DEMAC reproducibly enriches for chromatin components

**Fig. 1:**
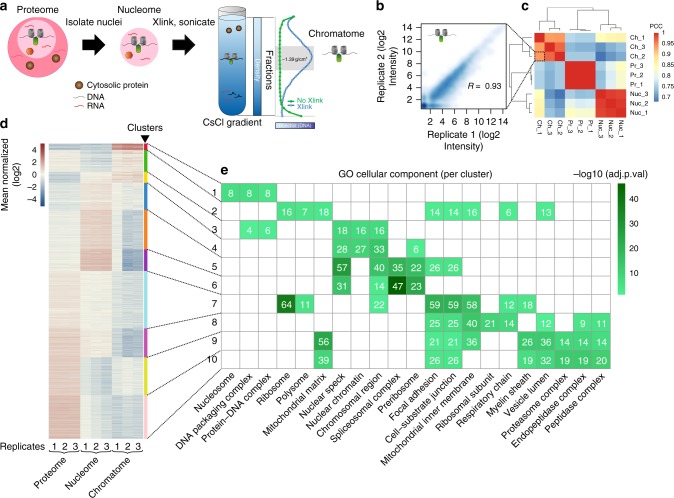
Strategy to quantify chromatin-associated proteins in different cellular states. **a** Experimental strategy for chromatin and nuclear enrichment. The blue and green lines to the right of the gradient represent Hoechst staining (*x*-axis) of different gradient fractions (*y*-axis). Xlink refers to cross-linking with formaldehyde. **b** Reproducibility between replicates of independent chromatome preparations. **c** Reproducibility among normalized Chromatome (Ch), Nucleome (Nuc), and cellular Proteome (Pr) signal. Scale is similarity as Pearson’s *R*. PCC Pearson’s correlation coefficient. **d** Clustering of signal at the protein level across subcellular fractions and all replicates. Scale is mean-normalized log2 converted reporter intensities. Colored annotation bar represents individual clusters. **e** Top two cellular component ontologies for each cluster and their representation in all clusters. Terms enriched in each cluster are noted on the *x*-axis, clusters on the *y*-axis. Number of proteins belonging to a category in a particular cluster are noted in white text for the corresponding cell. Significance is −log10 (adjusted *p-*value). White squares represent nonsignificant groups

To enrich for chromatin-bound proteins, we adapted a strategy based on the distinct buoyant density of cross-linked DNA–protein complexes in a cesium chloride (CsCl) gradient^21,22^. In brief, cells (here human T98G) were treated with formaldehyde, sonicated, adjusted to a high concentration of CsCl and subjected to high centrifugal force for at least 48 h, generating a stable isopycnic-density gradient (see Methods for extended protocol). Within this gradient, molecules migrate based on their buoyant density. While free proteins accumulate toward the top and free DNA and RNA toward the bottom, cross-linked nucleic-acid/protein complexes peak at a distinct density of 1.39 g/cm^3^ (Fig. 1a). Fractions with the desired density can thus be collected and CsCl removed by dialysis prior to further processing.

Importantly, the presence of DNA at the density of protein–DNA complexes of 1.39 g/cm^3^ is absolutely dependent on cross-linking (Fig. 1a, blue and green lines). Isolated fractions show strong enrichment of histones on an SDS–page gel (Supplementary Figure 1a) and upon western blotting for histone H4 as well as the chromatin-associated protein CTCF (Supplementary Figure 1b). As noted previously, RNaseA treatment before centrifugation^23,24^ reduces ribosomal signal and chromatin retention mediated via RNA binding, as can be seen for translation initiation factors (Supplementary Figure 1c).

Using this approach, we first asked if the gradient preparation results in an equal representation of the genome. To do so we sequenced DNA from both input and the chromatin fraction in duplicate, which revealed only minor differences in genomic representation at transcription start sites (TSS, Supplementary Figure 1d and e). The TSSs of transcribed genes display a localized small drop in read counts in the chromatin fraction (Supplementary Figure 1f, bottom), which is expected as nucleosome-free regions exist around active TSSs and can be recovered as free DNA from cross-linked chromatin, a feature that is utilized in the formaldehyde-assisted isolation of regulatory elements assay^25^.

Next, we asked for signs of experimental bias toward certain chromatin states. Toward this goal, we queried regions with different epigenetic marks. More specifically we analyzed existing ChIP-seq data for H3K36me3, H3K4me3, H3K27me3, H3K9me3 and H3K9me2 in Human SK–N–SH cells, a neuroblastoma line that is part of the ENCODE^26^ panel. This shows that DNA abundance in chromatin is strongly correlated with input, while showing only minimal correlations with all tested chromatin marks (Supplementary Figure 1g and h). We thus conclude that buoyant density-based fractionation recovers chromatin with no significant bias in regards to the local degree of heterochromatinization.

To investigate how DEMAC enriches for chromatin components, we contrasted it to the total proteome and nuclear proteome (nucleome) using standard procedures^27,28^. In brief, nuclear samples were obtained using NP-40 digestion of the cell membrane followed by washing of intact nuclei, and full proteome samples were processed by treating whole cells with the detergent RapiGest (Waters), boiled for 5 min followed by standard trypsin digestion conditions. Samples were prepared in triplicate, labeled with TMT and combined in a 9-plex experiment. High-resolution MS reproducibly quantified 3101 proteins, requiring a minimum of two unique peptides detected per protein and signal in all three replicates of one compartment (Fig. 1b, c, Supplementary Data 1). Comparison of the relative abundance of nuclear and cytoplasmic proteins between proteome, nucleome, and chromatome preparations revealed successive enrichment for known structural chromatin components such as histones and high-mobility group proteins (Supplementary Figure 1c). In contrast, proteins with higher cytosolic representation, such as ribosomes and translation initiation factors were sequentially depleted, demonstrating the differential representation of these protein groups in the proteome and chromatome samples.

To characterize enrichment of protein groups on a more global scale, we grouped proteins based on their relative abundance across the proteome, nucleome, and chromatome using affinity propagation clustering^29,30^. This clustering algorithm was used as it is deterministic, returning the number of clusters it finds within the data. We subsequently queried for enrichment of cellular component categories within each cluster^31^ (Fig. 1d, e). Groups with the highest enrichment in the chromatin fraction (clusters 1–3) include several chromatin components while those with highest signal in the nucleome (clusters 5 and 6) additionally contain splicing factors and proteins localized in nuclear specks. The clusters with highest signal in the full proteome (clusters 8–10), in contrast, were enriched for several classes of cytosolic proteins. Thus, DEMAC enables enrichment and detection of chromatin components in a reproducible manner.

### Differential chromatin binding across the cell cycle

We next applied DEMAC to query how stage-specific activities such as transcription, replication, and mitotic condensation are reflected in chromatome composition across the cell cycle. In particular, we sought to determine which regulatory proteins remain bound to metaphase chromosomes and thus could be involved in marking *cis*-acting sequences throughout M-phase.

**Fig. 2:**
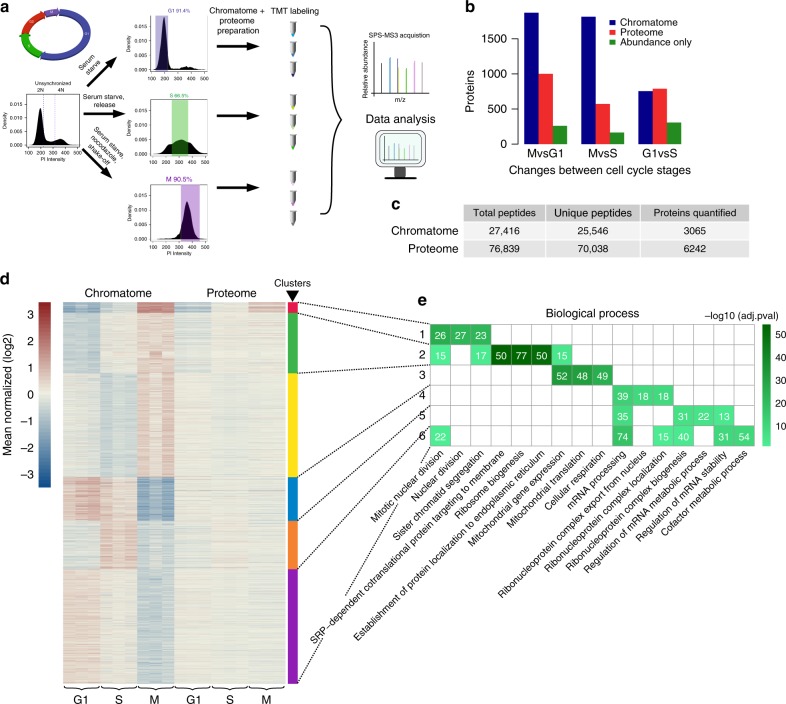
Changes in chromatin association across the cell cycle. **a** Outline of experimental procedure. Density plots represent propidium iodide staining of cellular DNA content (2N = diploid copy number). Cutoffs for G1, S, and G2/M are represented by blue, green, and purple colored boxes, respectively. Synchronization procedures are noted above arrows. **b** Total number of significantly changing proteins between cell cycle contrasts (*p* ≤ 0.001 and fold change ≥ 1.5, see Methods for significance determination), for proteome (red) and chromatome (blue) samples. Green bar represents those proteins where changes in chromatome correlate significantly with changes in proteome, and thus can be explained by protein abundance. **c** Number of proteins and peptides detected and quantified in proteome and chromatome. **d** Clustering of significantly changing chromatome proteins using affinity propagation clustering. Scale represents mean- normalized intensities after log2 transformation. Clusters are denoted by colored bars on the right with their respective numbers. **e** The top three biological process ontology terms associated with each cluster in **d** colored by −log10(adjusted *p-*value). Numbers in brackets represent number of proteins belonging to that category in the respective cluster as in 1e

Using the same human cell line, we enriched for cells in G1-, S-, and M-phase of the cell cycle by established synchronization techniques (Methods). This resulted in cell populations enriched for G1- (91.4%), S- (66.5%), or M-phase (90.5%), as determined by staining for DNA content (Fig. 2a). Additionally, phosphorylated threonine 11 of histone H3, a metaphase marker, was highly enriched in the mitotic cell preparation (Supplementary Figure 2a and b). Moreover, observed changes in cyclin abundance from full proteome measurements were in agreement with different cell cycle stages (Supplementary Figure 2c). Synchronized cells were subjected to chromatome and whole proteome analysis in triplicate, which were highly reproducible (Supplementary Figure 2d). The robustness of these measurements is further underscored by the reproducibility of quantification at the level of individual peptides as well as different peptides from the same protein (Supplementary Figure 2e). In total, 3065 proteins were quantified in the chromatin fractions and 6242 proteins in the total proteome samples (Fig. 2c, Supplementary Data 2).

To identify proteins that associate differentially with chromatin during the cell cycle, we selected those showing at least a significant 1.5-fold difference (*p* < 0.001, see Methods for significance determination) in enrichment between any pair of cell cycle phases (Fig. 2b, see Methods for significance determination). Using these criteria, 83.3% of proteins in the chromatome (2556 proteins) indeed change during the tested cell cycle phases. Clustering these proteins based on their change on chromatin across the cell cycle stages resulted in 6 distinct groups containing between 72 (cluster 1) and 768 (cluster 6) proteins (Fig. 2d). Clusters 4 and 6 are significantly depleted from mitotic chromatin and include many proteins involved in transcription, such as six subunits of RNA polymerase II as well as the PAF1 complex (Fig. 2d, e and Supplementary Figure 2e left), as expected given the global shutdown of transcription during mitosis^5^. Indeed, many splicing factors and RNA processing proteins were similarly depleted from M-phase chromatin (Fig. 2d, e, cluster 6). In contrast, clusters 2 and 3 contain several ribosomal and mitochondrial proteins. Signal for these proteins is increased in mitosis, and their binding may represent a portion of the protein mass previously suggested to coat mitotic chromosomes^32^.

In S-phase chromatin, factors are enriched that are involved in DNA replication, such as proteins of the replicative helicase, DNA polymerases as well as DNA ligase LIG1 (cluster 5, Supplementary Figure 2e middle). Several proteins involved in splicing were also enriched in this cluster, in line with findings that inhibition of the spliceosome can cause S-phase arrest^33^.

Finally, proteins that displayed highest signal in mitosis (cluster 1) contained factors critical for mitotic chromosome segregation. This includes all four members of the chromosome passenger complex (Supplementary Figure 2e right) as well as SMC proteins involved in mitotic nuclear division and MKI67, a protein that coats chromosomes during mitosis^34,35^. Based on these results, we conclude that our chromatome measurements recapitulate known large-scale chromatin associations during G1, S-phase, and mitosis.

### Disconnect between chromatin and proteome changes

For any given protein, the observed differences in binding to chromatin could either reflect differential association with chromatin, and thus regulated recruitment, or simply differences in protein abundance. To discriminate between these possibilities, we contrasted chromatome changes with those in the proteome. Using the same significance and fold change cutoff as above, we observe that ~16% of proteins change in abundance between G1 and mitosis, a percentage very close to previous work (19%) measuring ~3000 proteins^36^. More specifically, our analysis revealed that only ~15% of the variance in chromatin signal can be explained by coinciding changes in protein abundance (Fig. 2d and Supplementary Figure 3a). This general disconnect between chromatin binding and protein levels was highly reproducible (Supplementary Figure 3b). It is not due to limited detection of chromatin proteins in the full proteome, as 94% of the proteins quantified in the chromatome were also quantified in the proteome. Furthermore, the difference between binding and protein level is not caused by varying measurement sensitivities, as this relationship is consistent among the entire spectrum of protein intensities (Supplementary Figure 3c). We conclude that differential protein abundance between G1- and M-phase only accounts for a minority of observed changes on chromatin, while the majority is mediated via differential protein recruitment.

### Post-translational modifications during the cell cycle

**Fig. 3:**
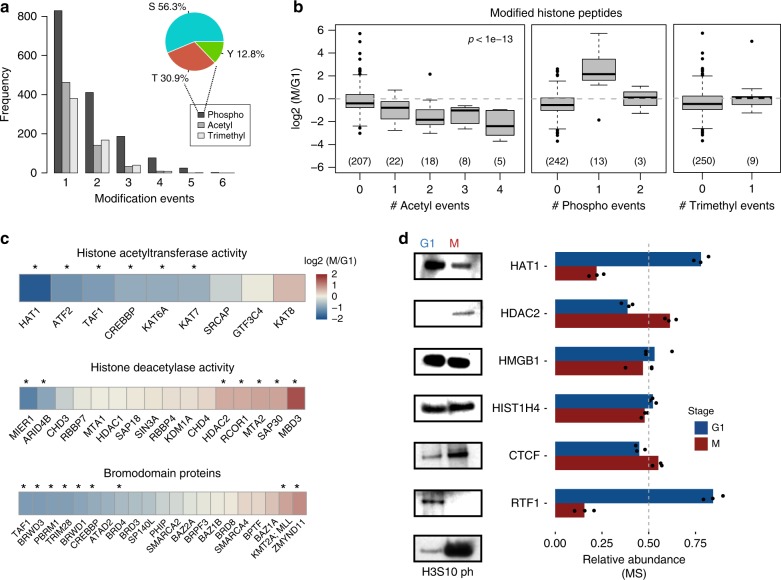
Differential abundance of post-translational modifications at the G1/M transition. **a** Frequency of peptides detected and quantified with given modifications. Pie-chart inset: percentage of S, T, and Y residues at class I phosphorylation sites (localization probability ≥ 0.7). **b** Changes in chromatome signal for all modified histone peptides between G1 and mitosis. Boxplots represent peptides either acetylated at lysine residues (left), phosphorylated at S, T, or Y residues (middle) or trimethylated at lysine residues (right). The box represents the middle 50% of the data, the line inside the box represents the median, and whiskers are defined by the most extreme values lying within 1.5 times the interquartile range. Outliers are shown as points. Numbers below boxes represent the number of peptides at each modification level. The *p-*value for slope of the fit of log2(M/G1) changes regressed against the number of modifications is noted in the upper right corner for acetylated peptides (see Methods). **c** Changes in signal for protein groups involved in writing (top), erasing (middle) and reading (bottom) histone acetylation. Asterisks above each heatmap cell represent changes that are significant (*p* < 0.001, see Methods for significance determination). **d** Western blot of G1 and M chromatin fractions (left) with corresponding signal from triplicate MS measurements (right)

**Fig. 4:**
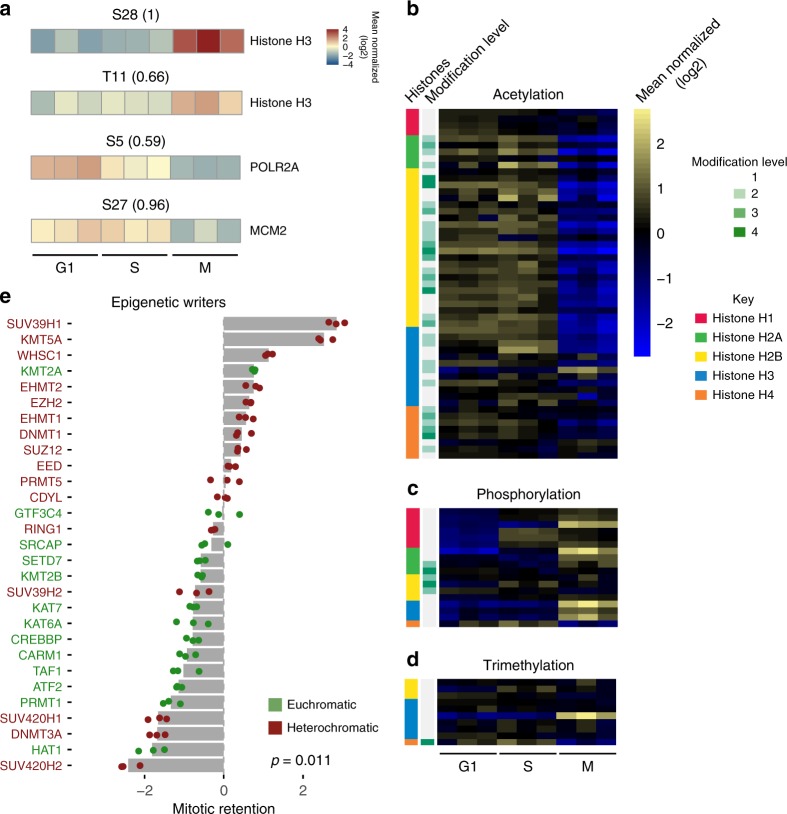
Differential binding of the epigenetic machinery during the cell cycle. **a** Changes in abundance for peptides in the chromatome. Top: Histone H3 phosphorylated at S28 or T11, Middle: RNA polymerase subunit POLR2A at serine 5 of the CTD repeats, Bottom: Serine 27 of MCM2. Numbers above denote the localization probabilities of phosphorylation. **b**–**d** Changes in histone acetylation (**b**), phosphorylation (**c**), and trimethylation (**d**) across cell cycle stages measured. The far-left annotation column denotes which histone the peptide was mapped to, the near left column (shades of green) denotes the number of modification events. Histone annotation has been simplified from possible isoforms for ease of interpretation. **e** Behavior of epigenetic writers between mitosis and G1. The *x*-axis is log2(M/G1) for each protein. Bars represent standard error of the mean. Red font represents proteins that tend to correlate with transcriptional repression, while proteins represented by green font represent proteins correlated with transcriptional activity. Significance between euchromatic and heterochromatic groups is noted on the bottom right (Wilcoxon rank-sum test)

Post-translational modifications of proteins, in particular phosphorylation, play a crucial regulatory role throughout the cell cycle^37^. Additionally, chromatin proteins such as histones are modified by acetylation or methylation at lysine residues and these modifications often correlate with distinct genomic activities^38^. Because chromatin substrates can be highly modified, we queried the chromatome data for peptides with phosphorylation of serine, threonine or tyrosine (STY) residues, as well as acetylation and trimethylation of lysines. In total, 1801 modified peptides were quantified, with a large proportion of these being phosphopeptides (1531), which seemed surprising given that additional enrichment steps are usually required before detection of this modification by MS (Supplementary Data 3). Over half of these phosphopeptides carry one modified residue, while the other peptides were multiply phosphorylated (Fig. 3a). Additionally, 646 peptides are acetylated and 596 are trimethylated. This includes known phosphorylation sites of RNA polymerase II (RPB1), MCM2 as well as threonine 11 of histone H3, a mark highly abundant in early mitosis (Fig. 4a) and that was used in determining the proportion of mitotic cells (Supplementary Figure 2b). Histone phosphorylation tends to increase during mitosis (Figs. 3b middle, 4c), while trimethylated histone peptides remained relatively unchanged (Figs. 3b right, 4d).

Histone acetylation in particular showed extensive regulation at the M/G1 transition, namely a significant loss of this mark during mitosis in agreement with previous findings (Fig. 3b left, 4b)^39,40^ and the known association of acetylated histone tail residues with ongoing transcription^38^. Notably, this trend is mirrored by overall depletion of modifiers and readers of this mark, while erasers are retained or enriched in mitosis. The nine proteins annotated with histone acetyltransferase (HAT) activity show overall depletion on mitotic chromatin (Fig. 3c, top). Similarly, bromodomain containing proteins capable of reading acetylated lysines show a trend toward depletion, although not statistically significant (Fig. 3c, bottom). In contrast to previous observations using immunofluorescence (IF)^39^, we observe retention and even enrichment for most of the factors involved in histone deacetylation (Fig. 3c, middle). Thus, histone acetylation changes closely align with the proteins responsible for writing, reading, and removing this epigenetic mark. Importantly the observed differential abundances for histone modifying enzymes as measured by MS are readily confirmed by Western Blotting for those proteins tested, as well as CTCF and the PAF1 member RTF1 (Fig. 3d).

Further exploration of epigenetic modifiers revealed a striking difference in retention between those functionally linked to euchromatin versus those linked to heterochromatin (p = 0.011, Wilcoxon rank-sum one-sided test) (Fig. 4e). The former tend to be depleted from mitotic chromatin and, in addition to HATs, include arginine and lysine methyltransferases such as PRMT1 and MLL4. In contrast, the H3K9 methyltransferases GLP, G9A and SUV39H1 as well as the maintenance DNA methyltransferase DNMT1 show increased binding to mitotic chromatin (Fig. 4e). Additionally, the three core subunits of PRC2 showed enriched signal on M-phase chromatin, suggesting retention of Polycomb in agreement with previous work in flies^41–43^ and mammalian cell lines^44^. Taken together, epigenetic modifiers associated with activation tend to be depleted during M-phase, while modifiers associated with gene repression are retained or even enriched. This functional selectivity in retention on condensed mitotic chromosomes implies a role for chromatin modifiers in the global shutdown of transcriptional activity during M-phase.

### Widespread retention of TFs on M-phase chromatin

**Fig. 5:**
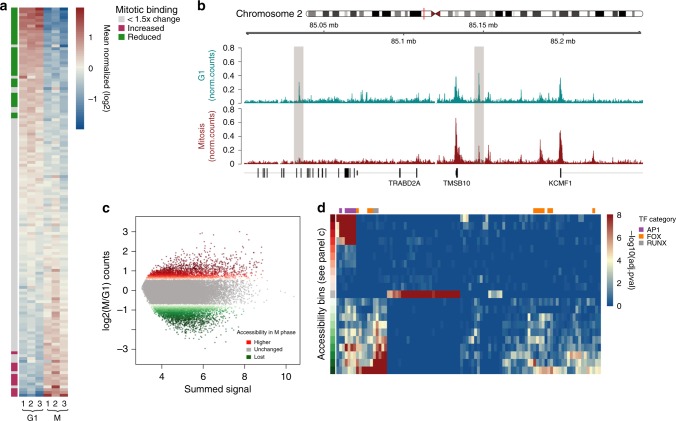
The regulatory landscape is largely retained on mitotic chromosomes. **a** Transcription factor binding dynamics between G1- and M-phase. Scale is mean normalized in log2 space. Factors significantly depleted in M-phase are indicated by the green annotation row and significantly enriched factors are denoted in red (*p* ≤ 0.001 and 1.5-fold change, see Methods for significance determination). **b** Screenshot of a 150 kb region, illustrating large scale conservation of the regulatory landscape. Shaded boxes represent local depletions in accessibility in mitosis. Scale is read counts normalized to library size. **c** Binning of distal peaks based on difference in accessibility between G1 and mitosis. Green and red points represent bins of reduced and increased accessibility in mitosis, respectively, while gray points represent unchanged peaks. **d** Enrichment of transcription factor motifs in bins shown in (c). Scale is −log10(adjusted *p-*value). Annotation column on top of the heatmap denotes transcription factors of the Fox and Runx families as well as AP1. Bins from panel (c) are represented by the annotation column on the far left

The fate of TFs during mitotic division has received considerable attention as a potential means to bookmark regions for subsequent gene activation. However, it has proven difficult to detect TFs by IF on condensed chromosomes likely due to epitope masking or denaturation^14,15,45^. Since cross-linking is reversed and detection relies on peptides, our MS-based approach overcomes these limitations. Indeed, we confidently quantify chromatin association for 137 TFs with at least two peptides despite the known overall low-expression levels of these proteins. This set encompasses DNA-binding domains from 37 different TF families (Fig. 5a and Supplementary Figure 4b)^46^. Of these 137 TFs, only ~24% (29) are depleted from mitotic chromatin (≥1.5-fold significant reduction, *p* < 0.001, see Methods for significance determination) while, strikingly, 42% show no difference or are even enriched on mitotic chromosomes (log2 (M/G1) ≥ 0). This extends recent studies of individual factors using live cell imaging, which revealed that several pluripotency TFs remain bound to mitotic chromatin^15,47,48^. Thus, comprehensive protein detection suggests that despite dramatic chromatin reorganization in mitosis, a large set of TFs remain bound, arguing that retention is not a discriminating feature of pluripotency factors. This also holds true if we include measurements from proteins represented by only a single peptide, which adds an additional 40 TFs (Supplementary Figure 4a and b) including five additional TF families based on DNA-binding domains. Importantly, they follow similar general trends as above, namely that most TFs remain associated with mitotic chromatin.

Overall, we find limited evidence that retention is a function of shared protein domains, with the exception of the forkhead box (FOX) family. Here, six members, of which three are quantified by multiple peptides, show consistent evidence of significant depletion (Supplementary Figure 4c). Notably, we did not obtain measurements for FoxA proteins with established pioneer activity that engage their binding sites in the context of nucleosomal DNA^49^.

In light of the widespread retention of several TFs with complex binding motifs, we next focused on general TFs (GTFs), which are involved in preinitiation complex formation, polymerase recruitment, and elongation. Consistent with previous observations using both FRAP and ChIP assays^50,51^, we observe that the TATA-Box binding protein, albeit only quantified by a single peptide, and several GTFs remain bound to mitotic chromatin (Supplementary Figure 4d). Our dataset now confirms this using chromatin proteomics and extends this to several other PIC members.

In contrast, GTFs associated with release from promoter proximal pausing (NELFA, NELFE, and SUPT5H) or transcriptional elongation proteins (PAF1 complex) show reduced presence on mitotic chromatin (Supplementary Figure 4d). This suggests that core promoter binding proteins are present on mitotic chromosomes, while factors involved in later stages of transcription are depleted. These findings agree with the observed lack of PolII in M-phase, and previous reports indicating that the elongation competent form of PolII appears in daughter nuclei subsequent to several GTFs upon entry into G1-phase^52^.

Taken together these observations reveal the continued presence of the majority of TFs and GTFs on mitotic chromosomes. As these proteins are known to occupy promoter and enhancer regions, this predicts that the chromatin regulatory landscape should be retained, for the most part, during M-phase.

### The regulatory landscape is largely maintained during mitosis

To determine whether TF persistence is reflected in the maintenance of regulatory regions, we measured chromatin accessibility in both G1- and M-phase genome-wide via ATAC-seq^53^, which was highly reproducible across replicates (Supplementary Figure 5a). Indeed, regulatory regions remain highly accessible in both cell cycle phases (Fig. 5b and Supplementary Figure 5b and c). While this conservation is most evident at TSSs, it is also true for most distal regions (Supplementary Figure 5c). This provides independent evidence for a large-scale retention of TFs and preinitiation complex members, as observed in the chromatome data (Fig. 5a and Supplementary Figure 4a and d). It is also in line with recent reports measuring chromatin accessibility during mitosis in both mouse and drosophila cells^15,54,55^.

To further relate TF presence in trans with chromatin accessibility *in cis*, we queried changes in accessibility of distal regulatory sites for enrichment of known TF motifs^56^ using HOMER^57^ (Fig. 5c, d and Supplementary Figure 5d). This revealed motifs enriched in distal peaks that lose accessibility, remain unchanged or have increased accessibility in mitosis. Importantly, these groups, even though only inferred by motif, recover FOX motifs within mitotically depleted peaks and AP1 motifs as present in mitotically enriched peaks, in line with the abundance changes of the respective TF families (Fig. 5d). This means that factor retention during mitosis can be related to changes in accessibility at distal regulatory elements and global maintenance of the regulatory landscape.

## Discussion

Differential association of proteins with chromatin is central to site-specific genome regulation. Here, we show that binding differences can be analyzed comprehensively using biophysical separation of formaldehyde cross-linked protein–DNA complexes combined with quantitative proteomics. This provides a valuable data source of differential chromatin association for proteins involved in transcription, replication, and division. Contrasting quantitative changes between mitotic and G1 enriched chromatin revealed a striking difference in selective mitotic retention of different chromatin pathways and widespread retention of TFs.

Several methods have been developed in recent years to interrogate the chromatin-bound proteome and have been discussed in recent reviews^58,59^. More pertinently, a few recent approaches have aimed to identify all proteins bound to chromatin in bulk, and these used xenopus extracts^60^, chicken cells^16,61^, or various human cell lines^17^. Due to differences in biological material, protein labeling, purification, and acquisition, it is inherently difficult to compare them quantitatively. We envision DEMAC to serve as a versatile addition as it can be performed from any cell or tissue used for ChIP. Importantly we show that DEMAC is highly reproducible, has no noticeable genomic bias and recapitulates well-described binding changes across cell cycle stages in a highly reproducible manner. We thus foresee it primarily as a tool for contrasting chromatin proteome changes between different cell types and/or genetic perturbations e.g. using CRISPR/Cas9.

While known protein components of chromatin are highly enriched in our preparation, it is challenging to assess the presence of potential contaminants^17^. It seems noteworthy in this context that nuclear lysis in DEMAC only occurs after formaldehyde inactivation, reducing the likelihood of cross-linking cytoplasmic proteins. Moreover, different to other protocols for chromatin purification, our density-based approach retains chromatin in solution throughout the preparation as it does not involve precipitation under conditions where chromatin is insoluble^24^.

The fact that we observe many post-translationally modified peptides not only illustrates that chromatin is differentially modified but also that formaldehyde cross-linking preserves such modifications. While DEMAC, like most proteomics approaches, requires many cells (~50 million), we foresee that this can be substantially decreased with increased sensitivity of MS instruments and sample preparation in smaller volumes.

Our study provides a rich data source for differential chromatin binding between the G1-, S-, and M-phase of the cell cycle. We have particularly focused our analysis on the difference between mitotic chromosomes and the G1-phase as the severe loss of transcriptional activity accompanied with chromosome condensation during mitosis represents a potential hurdle for faithful inheritance of gene expression patterns through cell division.

Recent elegant studies using live cell imaging already documented continuous binding to mitotic chromosomes of TFs required for pluripotency of embryonic stem cells. Tested factors included the pluripotency factors *Sox2*, *Oct4*, *Esrrb*, and *Klf4*, as well as *GATA1*, *FoxA1*, *Myc*, and *Rbpj*^15,47,48,62–65^. Additional evidence exists for RNA Pol I TFs^66^, while conflicting reports exist for several other factors^8^. Our study expands this observation now to many more factors regardless of function using comprehensive chromatin proteomics as an orthologous approach. Together this strongly argues that persistent binding to mitotic chromatin is a common feature of TFs, as has been observed before for pluripotency factors^15,48^.

While chromosomes condense 2–3-fold during mitosis^67^, our studies and others in mouse and drosophila suggest that this does not result in physical hindrance of TF binding, as most regulatory regions remain accessible^9,15,54,55^. In addition, while ultrastructural imaging of interphase and mitotic chromosomes revealed different packing densities between these cell cycle stages, the primary polymer structure of chromatin was relatively unchanged^68^. Taken together, accessibility, structure, and now our demonstration of persistent binding of TFs to mitotic chromatin suggest that mitotic retention is the rule rather than the exception. Our data further show that formaldehyde can efficiently crosslink TFs to mitotic chromosomes, arguing that cross-linking occurs at comparable efficiency in these cell cycle stages, and consequently seems unlikely to account for reported problems when imaging TFs under these conditions^15,45^.

The widespread conservation of the regulatory landscape and TF binding contrasts with both the loss of transcriptional activity and our observation of reduced presence of RNA polymerase as well as the PAF1 complex. Pertinent to this point, there is evidence that TFIIB binding is drastically reduced on mitotic chromosomes^69^, and that TAF3 binding to H3K4me3 is reduced by H3T3 phosphorylation in mitosis^70^. While we do not quantify either TFIIB or TAF3, we observe several other GTFs bound to mitotic chromatin. Thus the loss of TFIIB and specific TAFs such as TAF3 of the TFIID complex might account for strongly reduced transcription in mitosis, a process potentially coupled with the phosphorylation of general TFs^10^. Importantly, not only proteins involved in the process of transcription itself, but also those that mediate a chromatin structure permissive for transcription tend to be evicted from mitotic chromatin. It is tempting to speculate that the coinciding chromatin changes that we also observe for histone marks also contribute to the lack of transcription.

In line with such a model, proteins associated with establishing heterochromatin are retained suggesting a function in mitotic inheritance of silencing in these regions, which could even be functionally involved in chromosome condensation. The widespread conservation of TF binding at the protein level, as well as maintenance of the accessible regulatory landscape, is compatible with a model where binding functions in re-establishing transcriptional competence in interphase. One pertinent example of this is that we observe retention of the TF SP1 during mitosis, a TF that was previously thought to disengage mitotic chromatin^71^, but more recently was observed to remain using live cell imaging^15^. It is important to note that this general trend does not exclude more complex molecular scenarios for any given factor.

Regardless, our study and recent reports using Drosophila and mouse models^15,54,55^ establish that the regulatory landscape remains largely accessible on mitotic chromosomes. Here we readily explain these observations by showing actual protein retention on chromatin for many additional TFs, and furthermore implicate chromatin modification pathways in the propagation of chromatin states through mitosis.

## Methods

### Cell culture and cell cycle synchronization

T98G cells (origin human glioblastoma multiforme, ATCC^®^ CRL-1690^™^) were cultured at 37 °C and 7% CO_2_ in DMEM supplemented with 10% serum and 2 mM l-glutamine. Cells were synchronized in G1 using serum starvation for 72 h and S-phase cells were acquired by serum starving cells for 2 days, followed by a 22 h release in 10% serum, as described previously^72^. Mitotic cells were synchronized as previously described^73^ with slight modifications. Cells were first starved for 48 h, and then released into 20% serum containing media supplemented with 0.2 μg/mL nocodazole for 36 h. Mitotic cells were subsequently collected by shake-off.

### FACs analysis

For propidium iodide staining, T98G cells were trypsinized, washed in PBS and 70% ethanol was added dropwise while vortexing. Cells were incubated for a minimum of 30 min on ice, spun at 400×*g* for 5 min and washed twice with PBS. Cells were resuspended in 50 µl of 100 µg/mL RnaseA, incubated for 5–10 min at room temp, and then 200 µl of 50 µg/mL propidium iodide was added before passing through CellTrics^™^ 30 µM filters.

Analysis of mitotic cells for DNA content and H3T11 phosphorylation staining was performed as described^74^ except 25 µg/mL 7-AAD was used instead of propidium iodide. In brief, cells were fixed and washed as above, then resuspended in 500 µl PBS with 1% bovine serum albumin (BSA) and 0.4 µg of the H3T11 phospho antibody (Abcam ab5168). Cells were incubated 1 h at RT, washed once with 150 µl PBS + 1% BSA, and resuspended in PBS + 1% BSA and Alexa 488 donkey antimouse (Thermo Fisher A21202) diluted 1:300. Cells were incubated 30 min in the dark at RT, then spun as above and resuspended in 500 µl PBS with 10 µg/mL RnaseA and 25 µg/mL 7-AAD and passed through a CellTrics^™^ 30 µM filter.

Cells were acquired on a BD LSRII SORP Analyser (Beckton Dickson) using the BD FACSDiva 8.0.1 software. First gate FSC vs. SSC was used to exclude debris and dead cells. Doublets were excluded with FSC-W vs. FSC-H and SSC-W vs. SSC-H. An example of the gating strategy is shown in Supplementary Figure 6.

### Full proteome and nucleome preparation

Full proteome samples were extracted using the acid-cleavable surfactant RapiGest (Waters) as described^27^. Briefly, cells were lysed with RapiGest, incubated at 95° for 5 min, reduced and alkylated followed by overnight trypsin digestion and cleanup using Stage tips^75^. Nuclei were isolated using gentle NP-40 treatment (0.6%) for 3 min on ice, followed by centrifugation at 15×*g* in a cooled microcentrifuge (4 °C). Nuclei were washed twice in PBS, and then lysed using RapiGest as above for the full proteome.

### DEMAC

Nuclear preparation^76^ and CsCl fractionation^21^ were carried out as previously described with the following modifications:

Totally, 50–100 million cells were grown to ~70% confluency and washed twice with PBS. Cells were dissociated with trypsin, and trypsin was neutralized by adding fresh medium. The dissociated cells were then washed twice with PBS and resuspended in hypotonic buffer (10 mM HEPES pH 7.9, 1.5 mM MgCl_2_, 10 mM KCl, 0.5 mM DTT, and protease inhibitors) and incubated for 5 min at room temperature. Cells were then dounced with 10–15 strokes and subsequently spun for 5 min at 230×*g* in a precooled 4°C centrifuge. The nuclear pellet was then resuspended in 3 mL of buffer S1 (0.25 M sucrose, 10 mM MgCl_2_, and protease inhibitors), layered on top of a 3 mL cushion of buffer S3 (0.88 M sucrose, 0.5 mM MgCl_2_, and protease inhibitors) and spun for 10 min at 2800×*g* in a centrifuge precooled to 4 °C. Supernatant was removed and the pellet resuspended in 10 ml of fix buffer (50 mM HEPES pH 7.9, 1 mM EDTA, 0.5 mM EGTA, and 100 mM NaCl, 1% formaldehyde), and incubated for 10 min at RT with rotation. Formaldehyde was then quenched with 0.125 M Glycine and inverted 5 min at RT. Cross-linked cells were then spun down for 5 min at 600×*g* in a 4 °C cooled centrifuge and subsequently washed twice with ice cold PBS.

Cross-linked nuclei were washed once in sonication buffer (10 mM Tris pH 8, 1 mM EDTA, 0.5 mM EGTA, and protease inhibitors), resuspended in 3 mL sonication buffer and 0.5 g of glass beads were added. Chromatin was solubilized by sonication for three cycles on a Branson tip sonicator (30 s on, 15 s off at 20% power) while being cooled in a dry-ice/ethanol bath. Fresh protease inhibitor was added as well as 60 ng/mL RNaseA and tubes were rotated for 20–30 min at RT. Volume was adjusted to 4 mL with sonication buffer and sarkosyl added to a final concentration of 0.5%, and incubated with light shaking for 30 min @ RT. Insoluble material was removed by centrifugation at top speed in a precooled 4 °C microcentrifuge (12,000×*g*) for 10 min and supernatant transferred to a new tube. CsCl was added to a final density of 1.42 g/cm^3^ (~3.2 g in 4 mL) and spun in an ultracentrifuge (Beckman rotor SW55 Ti) at 186k×*g* for 48–72 h at RT.

After centrifugation, tubes were removed and two syringe needles were inserted just below the sarkosyl/lipid membrane formed at the top of the gradient. A third needle was inserted about 1 cm from the bottom of the tube and fractions collected in 1.5 mL microcentrifuge tubes (6 drops per tube, roughly ~150–300 μl per fraction). Totally, 100 μL from several fractions was measured to determine different densities in the gradient, and DNA content was measured by staining with Hoechst dye in a 96-well format. For this, 4 μL of each fraction was added to 200 μl of 1 μg/mL Hoechst dye, mixed and measured on a plate reader with 360 nM excitation and 460 nM emission. Relevant fractions were dialyzed using 3.5 KD MWCO dialysis tubing in 5 L of dialysis buffer (10 mM Tris pH 8, 5% Glycerol, 1 mM EDTA pH 8, and 0.5 mM EGTA) for 4–6 h. Samples were placed in 5 L of fresh dialysis buffer and allowed to dialyze overnight.

Dialyzed chromatin fractions were adjusted to 200 mM NaCl and incubated @ 95 °C for 20 min to reverse cross-links. Samples were then adjusted to 2 mM CaCl_2_, 1.5 mM MgCl_2_, 1× protease inhibitor (Roche, COEDTAF-RO), and 30 units of DnaseI were added and incubated for 10 min @ 37 °C to digest DNA. To precipitate protein, TCA was added to a final concentration of 20% and incubated on ice for 1 h. Samples were spun down for 30 min in a precooled microcentrifuge (4 °C) at max speed (12k×*g*), washed once with 10% TCA, once with 100% Acetone and allowed to dry @ RT. Dried samples were resuspended in 10 μl RCM buffer (0.5 M Tris pH 8.6, 6 M GnHCl) per 10 μg of protein, i.e., 50 μl for 50 μg of protein. Samples were adjusted to 16 mM TCEP, incubated for 30 min @ RT to reduce disulfide bonds, and subsequently alkylated by addition of 35 mM iodoacetamide and incubation for 30 min @ RT. Samples were diluted with 1.5 volumes of digestion buffer (50 mM Tris/HCl pH 8.6, 5 mM CaCl_2_), acetonitrile (ACN) was added to a final concentration of 5%, as well as Lys-C at a 50:1 ratio of protein/enzyme and incubated for 4 h at 37 °C. Samples were then diluted with one volume of digestion buffer and trypsin was added at a 50:1 ratio as above, and samples were incubated overnight at 37 °C. Subsequently samples were cleaned up on Stage tips^75^ and dried to be stored until further processing.

### MS sample preparation

Samples containing 25–50 μg of peptides were labeled with TMT 10-plex reagents (Thermo Fisher Scientific) as previously described^77^. In brief, 25–50 µg of peptides were resuspended in labeling buffer (2 M Urea/0.2 M HEPES, pH 8.3) for 5 min at RT with shaking (1400 rpm), and 6 µl of TMT reagent solution (0.2 mg TMT in DMSO) was added, vortexed and incubated 1 h at RT with shaking (500 rpm). To stop the reaction, 3 µl of 1.5 M hydroxylamine was added and samples were incubated 10 min at RT with shaking (500 rpm). Samples were then pooled and 10 µl of high-pH buffer (1 M potassium phosphate, pH 12 with NaOH) was added to each empty tube, pipette mixed, and added to the mixed peptide samples to increase yield. To acidify the mixture, 60 µl of 2 M HCl was then added, and subsequently 29.4 µl of 5% TFA to achieve a final concentration of 0.5% TFA. Peptides were then cleaned and desalted on StageTips^75^.

TMT labeled peptides were offline fractionated at high pH on a YMC Triart C18 0.5 × 250 mm column (YMC Europe GmbH) using the Agilent 1100 system (Agilent Technologies). Seventy-two fractions were collected for each experiment and concatenated into 12 or 24 fractions as previously described^78^. For each LC–MS run, approximately 1 μg of peptides were loaded onto a PepMap 100 C18 2 cm trap (Thermo Fisher) using the Proxeon EASY NanoLC-1000 system (Thermo Fisher). On-line peptide separation was performed on the 15 cm EASY-Spray C18 column (ES801, Thermo Fisher) by applying a linear gradient of increasing ACN concentration at a flow rate of 150 nL/min. An Orbitrap Fusion Tribrid (Thermo Fisher) mass spectrometer was operated in a data-dependent mode and TMT reporter ions were quantified using a synchronous precursor selection-based MS3 technology, as previously described^79^. In brief, the top 20 most intense precursor ions from the Orbitrap survey scan were selected for collision-induced dissociation (CID) fragmentation. The ion-trap analyzer was used to generate the MS2 CID spectrum from which the notches for the MS3 scan were selected. The MS3 spectrum was recorded using the Orbitrap analyzer at a resolution of 60,000.

### Proteomic data processing

Thermo MS raw data were searched against the human uniprot database (downloaded January 29, 2015) and a database of common contaminants with either Thermo Proteome Discoverer PD 2.1 (Thermo Scientific) and the Sequest HT search engine or MaxQuant (version 1.5.3.8) and the Andromeda search engine. The search parameters in PD were fully tryptic digestion, maximum of two missed cleavages, minimum peptide length of six amino acids, fixed carbamidomethyl modifications of cysteine as well as TMT6plex [+229 Da] of lysines and peptide N-termini; and oxidation of methionine as well as acetylation of the protein terminus as variable modifications. The maximum allowed mass tolerance for precursor ions measured in the Orbitrap was set to 10 ppm. The ion-trap fragment ion mass tolerance was set to 0.6 Da. Search parameters in MaxQuant^80^ were essentially the same as in PD, except up to 5 missed cleavages were allowed, and the mass tolerance for precursor ions was set to 20 ppm in a first-pass search prior to mass recalibration, followed by 4.5 ppm in the main search of the recalibrated data, and then the fragment ion mass tolerance was set to 0.5 Da. Protein and peptide identifications were filtered to a false-discovery rate of 0.01 based on the target-decoy search strategy^81^. For TMT MS3 reporter ion quantification the mass tolerance was set to 20 ppm in PD (0.1 Da in MaxQuant), only scans with average reporter ion s/n above 10 were used. Protein abundances were calculated based on the summed abundances of all unique and razor peptide reporter ion signals attributed to a protein.

For bioinformatic analyses the PD Proteins tables were exported to txt format, and the MaxQuant modificationSpecificPeptides.txt and Phospho(STY)Sites.txt table was used. To create a merged file from chromatin and proteome measurements in the cell cycle stages analyzed, Proteome Discoverer results for master proteins from the consensus merge were used to extract reporter intensities and peptide numbers from the individual searches. These proteins were then filtered for contaminants, proteins with at least two peptides quantified, and signal in all three replicates of at least one cell cycle stage. In cases where multiple proteins contained the same Entrez Gene ID, proteins were retained for the accession with highest summed reporter intensities in the proteome and chromatome. Proteins with no Entrez Gene ID were removed. Subsequently the reporter ion intensities for each channel were scaled down to the lowest signal reporter. The pseudocount of two was used as it was the smallest number that stabilized the mean-variance relationship of the reporter intensities. This pseudocount was added and the reporter intensities were then log2 transformed.

MaxQuant peptide search results were first filtered for reverse, contaminant, and proteins with no gene name. Modified peptides were first filtered on an Andromeda score ≥ 40 and delta score ≥ 8 and then filtered to have quantitative information for at least all three replicates of one cell cycle stage. Intensities were normalized using the VSN package in R as has been described for isobarically labeled peptides^82^.

### Determination of significant changes

To calculate significance of changes between multiple contrasts, a limma-trend approach was used^83^. In short, fold changes and standard errors were estimated by fitting a linear model on the log2-transformed values for each protein using the lmFit function. Standard errors were then smoothed using empirical Bayes with the function eBayes. In this function, the argument trend was set to TRUE in order to take into account the slightly nonconstant mean-variance relationship. The resulting *p* values were adjusted using the FDR approach^84^. Significantly changing proteins were defined to be all proteins with an adjusted *p* value ≤ 0.001 and a log2 fold change of at least 1.5 in any given contrast. For Fig. 3b, a robust regression of log2 fold changes in chromatin signal between M and G1 against the number of acetylated residues was carried out using the R function rlm from the MASS package^85^. To determine the significance of the slope, a Wald test was carried out using the function f.robftest from the sfsmisc package^86^. For enrichment of TF families, three different gene-set enrichment algorithms from the limma package were used (mroast, camera, and romer^87–89^).

### Clustering of proteins and GO enrichment

Reporter ion intensities from significantly changing proteins were clustered based on mean normalized values for the nine replicates measured for each protein. Clustering was carried out using affinity propagation clustering^29^ implemented in the R package apcluster^30^. GO annotation was subsequently performed on the clustered proteins using the clusterProfiler package^31^. Significance of terms was calculated based on a hypergeometric distribution and *p* values were corrected using the Benjamini–Hochberg^84^ method by setting the parameter *p*AdjustMethod = BH.

### Chromatin fraction sequencing

DNA from input, chromatin and top fractions in the CsCl gradient was first incubated with 0.2 mg/ml RNaseA and 50 μg/ml Proteinase K (20 μg/ml) and incubated for 2 h at 55 °C. Sodium dodecyl sulphate (SDS) and NaCl were subsequently added to final concentrations of 1% and 100 mM, respectively, and the samples decross-linked by overnight incubation at 60 °C. DNA was cleaned using phenol chloroform/ethanol precipitation, and sonicated to ~300 bp in size using Covaris as per the manufacturer’s recommendations. Libraries were constructed from these populations and sequenced on the HiSeq platform (Illumina) with 50 bp single-end reads.

### Western blotting

For each sample, approximately 10 µg of protein from TCA precipitated chromatin samples were boiled for 10 min in Novex^®^ loading buffer, and run on Novex^®^ 4–12% Bis/Tris gels using MES buffer. Proteins were transferred to PVDF membranes using the Novex^®^ system, blocked for 1–2 h with 5% milk in TBST and incubated with the respective antibodies overnight at 4 °C in 5% milk in TBST. Antibody dilutions are as follows: Histone H4 (Abcam ab134212) @ 1:1000, CTCF (Abcam ab128873) @ 1:2000, HAT1 (Thermo PA5-57817) @ 1:1000, RTF1 (Proteintech 12170-1-AP) @ 1:1000, HDAC2 (Abcam ab32117) @ 1:2000, HMGB1 (Abcam ab18256) @ 1:1000, Histone H3 Phospho S10 (Abcam ab14955) @ 1:1000. For uncropped western blots with molecular weight markers, please see Supplementary Figure 7.

### ENCODE data processing

ChIP data was downloaded directly from the ENCODE^26^ website and aligned with default parameters using the QuasR^90^ package in R. Genome tiling was done using the GenomicRanges^91^ package in R, and subsequently read counts were tallied using QuasR.

### ATAC-seq processing and analysis

ATAC-seq of cells synchronized in G1- and M-phase was carried out as described^53^. Adapters were first filtered using the cutadapt software^92^, and one single base pair was trimmed from the 3′ end of both reads to allow for mapping of overlapping reads. Reads were mapped to the hg19 build of the human genome using Rbowtie in the QuasR package with the modified alignment parameters: “-m 3 -k 1 --best --strata --maxins 2000 --tryhard”. Mitochondrial reads were subsequently removed using samtools. Peaks were called on merged replicates using macs2^93^ and the following command arguments (--nomodel --broad --keep-dup all). Peaks in G1 and mitosis were then merged for downstream analysis. For motif enrichments in distal peaks, read counts in peaks were scaled down to the smallest library size, and these counts were used to determine fold changes in accessibility between mitosis and G1. These peaks were ranked by accessibility change and separated into 21 bins. The top 10 bins that gained accessibility in mitosis as well as the bottom 10 that lost accessibility contained 500 peaks each. The middle bin contained all other distal peaks (see Fig. 5c). Homer was used to determine enrichment of TF motifs within each bin compared to all other bins. Only TFs that are expressed in T98G cells were used, determined based on published RNA-seq data in this cell line^94^. For this analysis, PWMs from the 2016 Jaspar^56^ release were used. The resulting *p* values were subsequently adjusted using the Benjamini–Hochberg^84^ approach, and all TFs that had at least one significant bin were kept.

### Code availability

All *R* scripts used in data analysis and generation of figures are available upon request.

## Electronic supplementary material

Supplementary Information

Description of Additional Supplementary Files

Supplementary Data 1

Supplementary Data 2

Supplementary Data 3

## Data Availability

Proteomics data were deposited in the PRIDE database with the accession code PXD008033. Sequencing data were deposited in GEO with the accession code GSE106482. All other data supporting the findings of this study are available from the corresponding author upon reasonable request.

## References

[1] Antonin W, Neumann H (2016). Chromosome condensation and decondensation during mitosis. Curr. Opin. Cell Biol..

[2] Hustedt N, Durocher D (2016). The control of DNA repair by the cell cycle. Nat. Cell Biol..

[3] Ruijtenberg S, van den Heuvel S (2016). Coordinating cell proliferation and differentiation: Antagonism between cell cycle regulators and cell type-specific gene expression. Cell Cycle.

[4] Siddiqui K., On K. F., Diffley J. F. X. (2013). Regulating DNA Replication in Eukarya. Cold Spring Harbor Perspectives in Biology.

[5] Parsons GG, Spencer CA (1997). Mitotic repression of RNA polymerase II transcription is accompanied by release of transcription elongation complexes. Mol. Cell Biol..

[6] Michelotti EF, Sanford S, Levens D (1997). Marking of active genes on mitotic chromosomes. Nature.

[7] Wang F, Higgins JM (2013). Histone modifications and mitosis: countermarks, landmarks, and bookmarks. Trends Cell Biol..

[8] Kadauke S, Blobel GA (2013). Mitotic bookmarking by transcription factors. Epigenetics Chromatin.

[9] Martinez-Balbas MA, Dey A, Rabindran SK, Ozato K, Wu C (1995). Displacement of sequence-specific transcription factors from mitotic chromatin. Cell.

[10] Gottesfeld JM, Forbes DJ (1997). Mitotic repression of the transcriptional machinery. Trends Biochem. Sci..

[11] John S, Workman JL (1998). Bookmarking genes for activation in condensed mitotic chromosomes. Bioessay.

[12] Rizkallah R, Hurt MM (2009). Regulation of the transcription factor YY1 in mitosis through phosphorylation of its DNA-binding domain. Mol. Biol. Cell.

[13] Chuang LS (2016). Aurora kinase-induced phosphorylation excludes transcription factor RUNX from the chromatin to facilitate proper mitotic progression. Proc. Natl Acad. Sci. USA.

[14] Lerner J (2016). Human mutations affect the epigenetic/bookmarking function of HNF1B. Nucleic Acids Res..

[15] Teves, S. S. et al. A dynamic mode of mitotic bookmarking by transcription factors. *eLife* **5**, 10.7554/eLife.22280 (2016). 10.7554/eLife.22280 PMC5156526 27855781

[16] Ohta S (2010). The protein composition of mitotic chromosomes determined using multiclassifier combinatorial proteomics. Cell.

[17] Kustatscher G (2014). Proteomics of a fuzzy organelle: interphase chromatin. EMBO J..

[18] Landt SG (2012). ChIP-seq guidelines and practices of the ENCODE and modENCODE consortia. Genome Res..

[19] Satterlee JS, Schubeler D, Ng HH (2010). Tackling the epigenome: challenges and opportunities for collaboration. Nat. Biotechnol..

[20] Thompson A (2003). Tandem mass tags: a novel quantification strategy for comparative analysis of complex protein mixtures by MS/MS. Anal. Chem..

[21] Orlando V, Strutt H, Paro R (1997). Analysis of chromatin structure by in vivo formaldehyde cross-linking. Methods.

[22] Solomon MJ, Larsen PL, Varshavsky A (1988). Mapping protein–DNA interactions in vivo with formaldehyde: evidence that histone H4 is retained on a highly transcribed gene. Cell.

[23] Jackson V (1999). Formaldehyde cross-linking for studying nucleosomal dynamics. Methods.

[24] Kustatscher G, Wills KL, Furlan C, Rappsilber J (2014). Chromatin enrichment for proteomics. Nat. Protoc..

[25] Giresi PG, Kim J, McDaniell RM, Iyer VR, Lieb JD (2007). FAIRE (formaldehyde-assisted isolation of regulatory elements) isolates active regulatory elements from human chromatin. Genome Res..

[26] Consortium EP (2012). An integrated encyclopedia of DNA elements in the human genome. Nature.

[27] Klimmeck D (2012). Proteomic cornerstones of hematopoietic stem cell differentiation: distinct signatures of multipotent progenitors and myeloid committed cells. Mol. Cell. Proteom..

[28] Teves SS, Henikoff S (2012). Salt fractionation of nucleosomes for genome-wide profiling. Methods Mol. Biol..

[29] Frey BJ, Dueck D (2007). Clustering by passing messages between data points. Science.

[30] Bodenhofer U, Kothmeier A, Hochreiter S (2011). APCluster: an R package for affinity propagation clustering. Bioinformatics.

[31] Yu G, Wang LG, Han Y, He QY (2012). clusterProfiler: an R package for comparing biological themes among gene clusters. Omics.

[32] Booth DG (2016). 3D-CLEM Reveals that a major portion of mitotic chromosomes is not chromatin. Mol. Cell.

[33] Karamysheva Z, Diaz-Martinez LA, Warrington R, Yu H (2015). Graded requirement for the spliceosome in cell cycle progression. Cell Cycle.

[34] Saiwaki T, Kotera I, Sasaki M, Takagi M, Yoneda Y (2005). In vivo dynamics and kinetics of pKi-67: transition from a mobile to an immobile form at the onset of anaphase. Exp. Cell Res..

[35] Verheijen R (1989). Ki-67 detects a nuclear matrix-associated proliferation-related antigen. II. Localization in mitotic cells and association with chromosome. J. Cell Sci..

[36] Dephoure N (2008). A quantitative atlas of mitotic phosphorylation. Proc. Natl Acad. Sci. USA.

[37] Nigg EA (2001). Mitotic kinases as regulators of cell division and its checkpoints. Nat. Rev. Mol. Cell Biol..

[38] Kouzarides T (2007). Chromatin modifications and their function. Cell.

[39] Kruhlak MJ (2001). Regulation of global acetylation in mitosis through loss of histone acetyltransferases and deacetylases from chromatin. J. Biol. Chem..

[40] McManus KJ, Hendzel MJ (2006). The relationship between histone H3 phosphorylation and acetylation throughout the mammalian cell cycle. Biochem. Cell Biol..

[41] Follmer NE, Wani AH, Francis NJ (2012). A polycomb group protein is retained at specific sites on chromatin in mitosis. PLoS Genet..

[42] Fonseca JP (2012). In vivo Polycomb kinetics and mitotic chromatin binding distinguish stem cells from differentiated cells. Genes Dev..

[43] Fanti L (2008). The trithorax group and Pc group proteins are differentially involved in heterochromatin formation in Drosophila. Chromosoma.

[44] Aoto T, Saitoh N, Sakamoto Y, Watanabe S, Nakao M (2008). Polycomb group protein-associated chromatin is reproduced in post-mitotic G1-phase and is required for S-phase progression. J. Biol. Chem..

[45] Pallier C (2003). Association of chromatin proteins high-mobility group box (HMGB) 1 and HMGB2 with mitotic chromosomes. Mol. Biol. Cell.

[46] Zhang HM (2012). AnimalTFDB: a comprehensive animal transcription factor database. Nucleic Acids Res..

[47] Festuccia N (2016). Mitotic binding of Esrrb marks key regulatory regions of the pluripotency network. Nat. Cell Biol..

[48] Liu Y (2017). Widespread mitotic bookmarking by histone marks and transcription factors in pluripotent stem cells. Cell Rep..

[49] Iwafuchi-Doi M, Zaret KS (2014). Pioneer transcription factors in cell reprogramming. Genes Dev..

[50] Chen D, Hinkley CS, Henry RW, Huang S (2002). TBP dynamics in living human cells: constitutive association of TBP with mitotic chromosomes. Mol. Biol. Cell.

[51] Christova R, Oelgeschlager T (2002). Association of human TFIID-promoter complexes with silenced mitotic chromatin in vivo. Nat. Cell Biol..

[52] Prasanth KV, Sacco-Bubulya PA, Prasanth SG, Spector DL (2003). Sequential entry of components of the gene expression machinery into daughter nuclei. Mol. Biol. Cell.

[53] Buenrostro, J. D., Wu, B., Chang, H. Y. & Greenleaf, W. J. ATAC-seq: A method for assaying chromatin accessibility genome-wide. *Curr. Protoc. Mol. Biol.* **109**, 21–29, 10.1002/0471142727.mb2129s109 (2015). 10.1002/0471142727.mb2129s109 PMC4374986 25559105

[54] Blythe, S. A. & Wieschaus, E. F. Establishment and maintenance of heritable chromatin structure during early Drosophila embryogenesis. *eLife* **5**, 10.7554/eLife.20148 (2016). 10.7554/eLife.20148 PMC5156528 27879204

[55] Hsiung CC (2015). Genome accessibility is widely preserved and locally modulated during mitosis. Genome Res..

[56] Mathelier A (2016). JASPAR 2016: a major expansion and update of the open-access database of transcription factor binding profiles. Nucleic Acids Res..

[57] Heinz S (2010). Simple combinations of lineage-determining transcription factors prime cis-regulatory elements required for macrophage and B cell identities. Mol. Cell.

[58] Noberini R, Sigismondo G, Bonaldi T (2016). The contribution of mass spectrometry-based proteomics to understanding epigenetics. Epigenomics.

[59] Wierer M, Mann M (2016). Proteomics to study DNA-bound and chromatin-associated gene regulatory complexes. Hum. Mol. Genet..

[60] Raschle M (2015). DNA repair. Proteomics reveals dynamic assembly of repair complexes during bypass of DNA cross-links. Science.

[61] Ohta S (2016). Proteomics analysis with a nano random forest approach reveals novel functional interactions regulated by SMC complexes on mitotic chromosomes. Mol. Cell Proteom..

[62] Deluz C (2016). A role for mitotic bookmarking of SOX2 in pluripotency and differentiation. Genes Dev..

[63] Kadauke S (2012). Tissue-specific mitotic bookmarking by hematopoietic transcription factor GATA1. Cell.

[64] Caravaca JM (2013). Bookmarking by specific and nonspecific binding of FoxA1 pioneer factor to mitotic chromosomes. Genes Dev..

[65] Lake RJ, Tsai PF, Choi I, Won KJ, Fan HY (2014). RBPJ, the major transcriptional effector of Notch signaling, remains associated with chromatin throughout mitosis, suggesting a role in mitotic bookmarking. PLoS Genet..

[66] Chen D (2005). Condensed mitotic chromatin is accessible to transcription factors and chromatin structural proteins. J. Cell Biol..

[67] Raccaud M, Suter DM (2018). Transcription factor retention on mitotic chromosomes: regulatory mechanisms and impact on cell fate decisions. FEBS Lett..

[68] Ou Horng D., Phan Sébastien, Deerinck Thomas J., Thor Andrea, Ellisman Mark H., O’Shea Clodagh C. (2017). ChromEMT: Visualizing 3D chromatin structure and compaction in interphase and mitotic cells. Science.

[69] Liang K (2015). Mitotic transcriptional activation: clearance of actively engaged Pol II via transcriptional elongation control in mitosis. Mol. Cell.

[70] Varier RA (2010). A phospho/methyl switch at histone H3 regulates TFIID association with mitotic chromosomes. EMBO J..

[71] He S, Davie JR (2006). Sp1 and Sp3 foci distribution throughout mitosis. J. Cell Sci..

[72] Takahashi Y, Rayman JB, Dynlacht BD (2000). Analysis of promoter binding by the E2F and pRB families in vivo: distinct E2F proteins mediate activation and repression. Genes Dev..

[73] Song B, Liu XS, Davis K, Liu X (2011). Plk1 phosphorylation of Orc2 promotes DNA replication under conditions of stress. Mol. Cell Biol..

[74] Zhu, H. Protocol to Determine Mitotic Index by FACS. *Bio-Protocol* **2**, e196 (2012).

[75] Rappsilber J, Mann M, Ishihama Y (2007). Protocol for micro-purification, enrichment, pre-fractionation and storage of peptides for proteomics using StageTips. Nat. Protoc..

[76] Sara ten Have, K. H., Angus I. Lammond. in *Functional Genomics* (Germana Meroni ed) Ch. 9, (InTech, 2012).

[77] Ahrne E (2016). Evaluation anD Improvement of Quantification Accuracy in Isobaric Mass Tag-based Protein Quantification Experiments. J. Proteome Res..

[78] Wang Y (2011). Reversed-phase chromatography with multiple fraction concatenation strategy for proteome profiling of human MCF10A cells. Proteomics.

[79] McAlister GC (2014). MultiNotch MS3 enables accurate, sensitive, and multiplexed detection of differential expression across cancer cell line proteomes. Anal. Chem..

[80] Cox J, Mann M (2008). MaxQuant enables high peptide identification rates, individualized p.p.b.-range mass accuracies and proteome-wide protein quantification. Nat. Biotechnol..

[81] Elias JE, Gygi SP (2010). Target-decoy search strategy for mass spectrometry-based proteomics. Methods Mol. Biol..

[82] Karp NA (2010). Addressing accuracy and precision issues in iTRAQ quantitation. Mol. Cell Proteom..

[83] Law CW, Chen Y, Shi W, Smyth GK (2014). voom: Precision weights unlock linear model analysis tools for RNA-seq read counts. Genome Biol..

[84] Benjamini Y, Hochberg Y (1995). Controlling the false-discovery rate—a practical and powerful approach to multiple testing. J. R. Stat. Soc. B Methods.

[85] Venables W. N., Ripley B. D. (2002). Modern Applied Statistics with S.

[86] Maechler, M. et al. sfsmisc: Utilities from ‘Seminar fuer Statistik’ ETH Zurich. *R package version 1.1-1* (2017).

[87] Wu D (2010). ROAST: rotation gene-set tests for complex microarray experiments. Bioinformatics.

[88] Wu D, Smyth GK (2012). Camera: a competitive gene-set test accounting for inter-gene correlation. Nucleic Acids Res..

[89] Ritchie ME (2015). limma powers differential expression analyses for RNA-sequencing and microarray studies. Nucleic Acids Res..

[90] Gaidatzis D, Lerch A, Hahne F, Stadler MB (2015). QuasR: quantification and annotation of short reads in R. Bioinformatics.

[91] Lawrence M (2013). Software for computing and annotating genomic ranges. PLoS Comput. Biol..

[92] Martin Marcel (2011). Cutadapt removes adapter sequences from high-throughput sequencing reads. EMBnet.journal.

[93] Zhang Y (2008). Model-based analysis of ChIP-Seq (MACS). Genome Biol..

[94] Shraibman B, Kadosh DM, Barnea E, Admon A (2016). Human leukocyte antigen (HLA) peptides derived from tumor antigens induced by inhibition of DNA methylation for development of drug-facilitated immunotherapy. Mol. Cell Proteom..

