# Supplementary material for: Cell cycle-resolved chromatin proteomics reveals the extent of mitotic preservation of the genomic regulatory landscape
Source: Nat Commun. 2018 Oct 2;9:4048. doi: 10.1038/s41467-018-06007-5 (PMC6168604; doi:10.1038/s41467-018-06007-5)
Supplement: Supplementary file 1 — Supplementary Information [file 41467_2018_6007_MOESM1_ESM.pdf]

Cell cycle-resolved chromatin proteomics reveals the extent of mitotic preservation of the genomic regulatory landscape

Ginno et al.

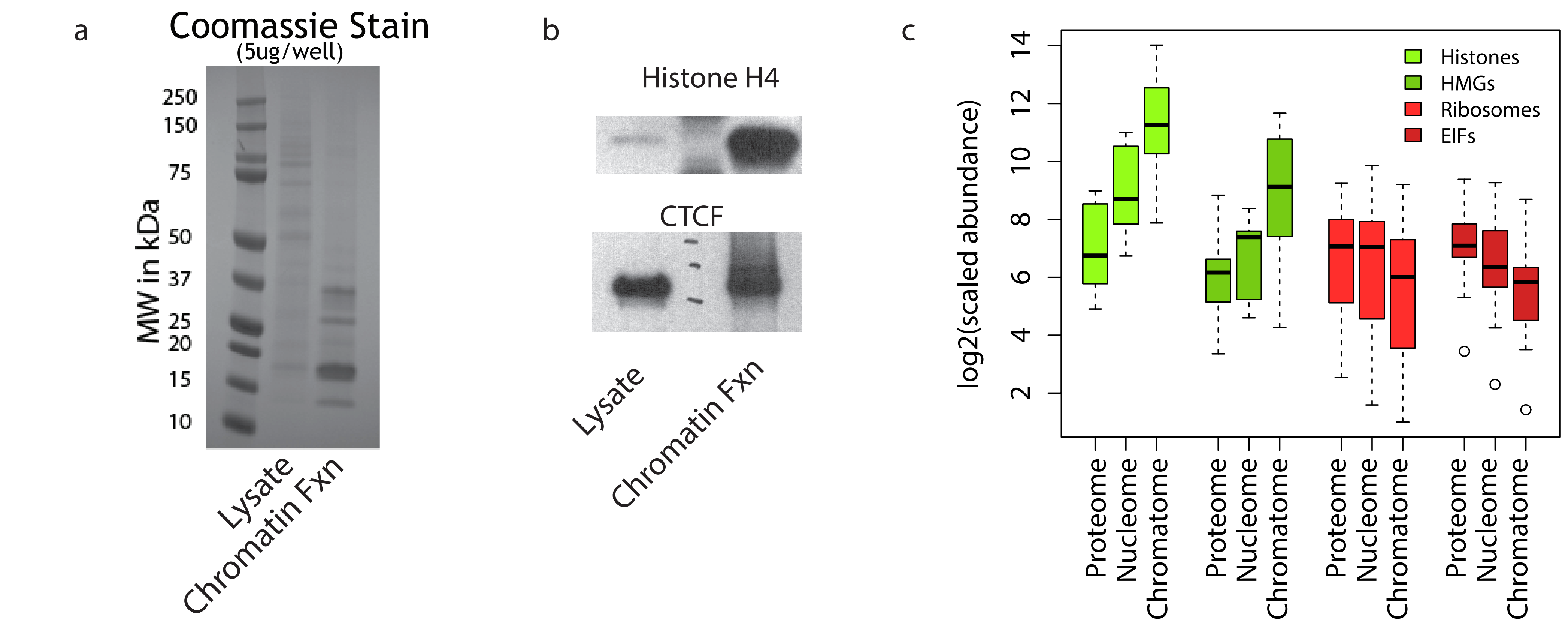

Supplementary Figure 1 Chromatin enrichment and assessment of genomic bias. **a** Coomassie staining of protein from a whole cell lysate and chromatin fraction (5ug in each lane). **b** Western blot for histone H4 (Abcam ab134212) and CTCF (Abcam ab128873) in the chromatin fraction and whole cell lysate. **c** Boxplot of summed normalized intensities for histones, hmgs, ribosomes and translation initiation factors (eifs) in each cellular compartment (Proteome, Nucleome and Chromatome resp.). The box represents the middle 50% of the data, the line inside the box represents the median, and whiskers are defined by the most extreme values lying within 1.5 times the interquartile range. **d** Left, Outline of sequencing samples to test for overt bias in genomic representation, Right, agarose gel of DNA fragment sizes from Input chromatin and the gradient chromatin fraction. **e** Scatterplot of log2(scaled read counts) in 2kb genomic tiles for Input and the chromatin fraction. **f** Metaplot of read counts surrounding TSS centered on max cage signal (FANTOM consortium) for Input (top) and chromatin fraction (bottom). Promoters were binned on RPKM values (light blue = lowest, dark blue = highest). Legend denotes max RPKM per bin. **g** Correlation of read counts from input, chromatin fraction and top fraction DNA as well as 5 histone marks (SK-N-SH data from ENCODE) in 2kb genomic tiles. **h** Chromosome 19 tiled into 2kb bins with signal for Input, chromatin and histone marks noted. Read counts were scaled to smallest library size and the number of reads/mappable kb are plotted. The y-axis scale from the input panel (top panel) is identical for all other panels.

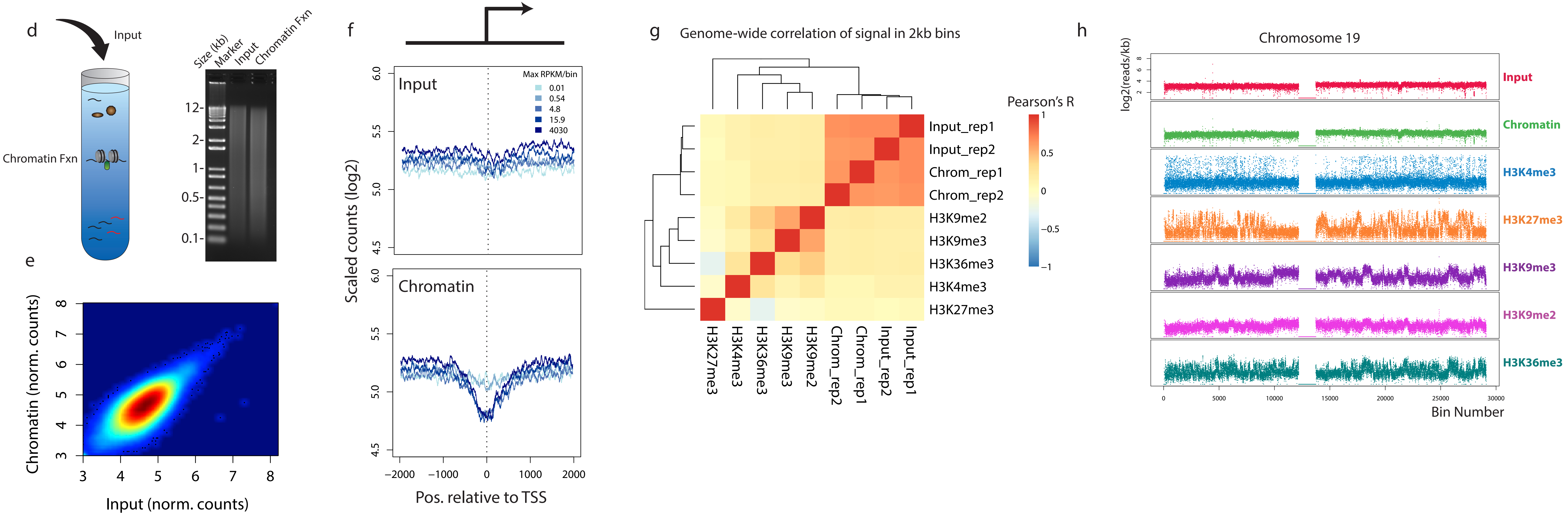

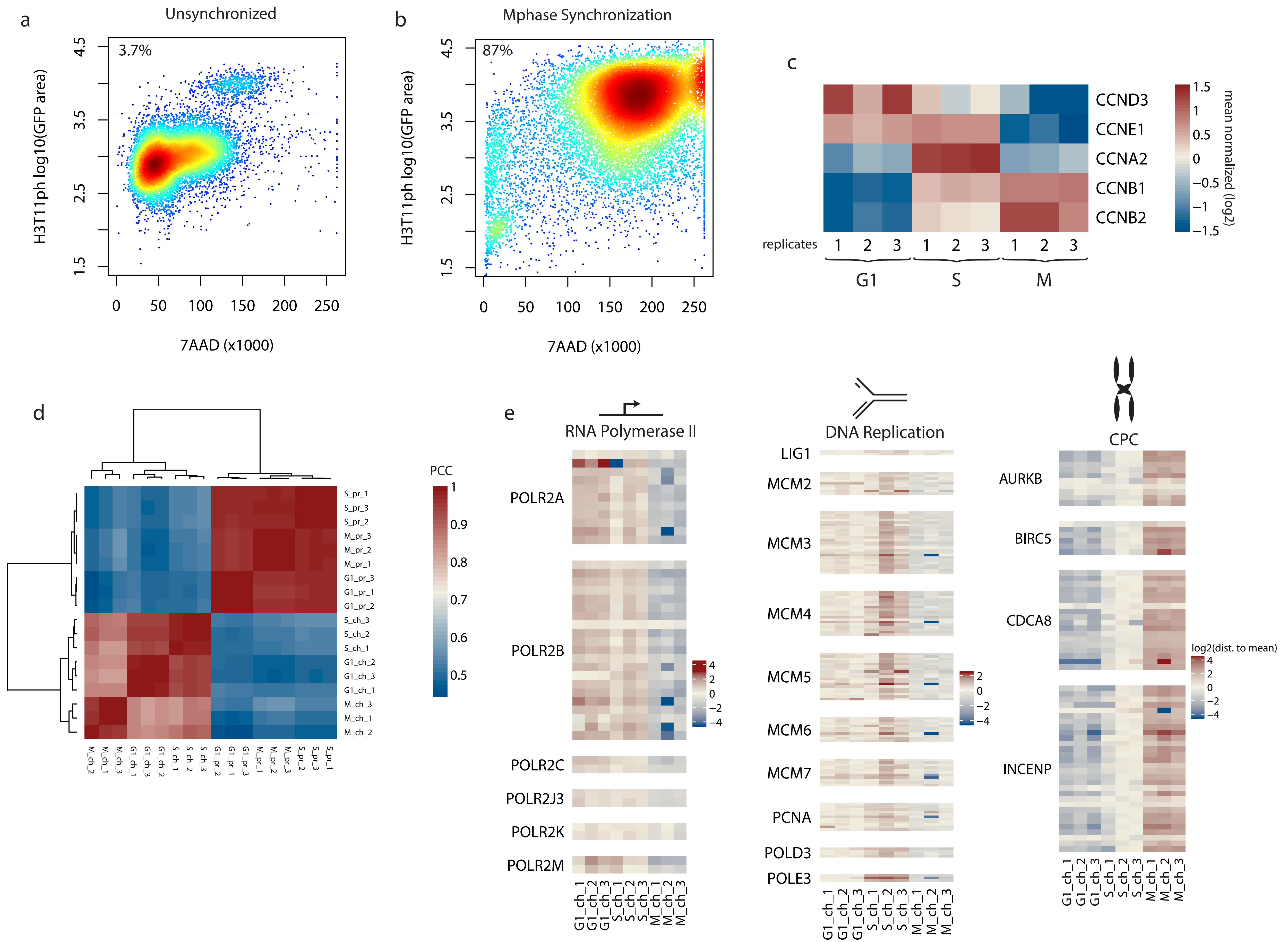

Supplementary Figure 2 Synchronization efficacy and reproducibility of chromatin measurements. **a** 7-AAD and H3S10 FACs sorting of unsynchronized cells, M-phase cells are the top right compartment. ~10,000 cells were measured in each plot. Number in the upper left corner represents proportion of mitotic cells. **b** Same as in panel a but for M-phase synchronized cells. **c** Cyclin changes in full proteome samples. Scale is in mean normalized log2(reporter intensities). **d** Correlation of normalized intensities for chromatome (ch) and proteome (pr) measurements for the synchronized cell stages. **e** Heatmaps of quantified peptides for the respective proteins. RNA polymerase II components are far left, members of the replisome in the middle and the chromosome passenger complex is depicted far right. Scale is noted on the legend. Outlier values greater than 4 in log2 space were scaled down to 4 to allow better visualization.

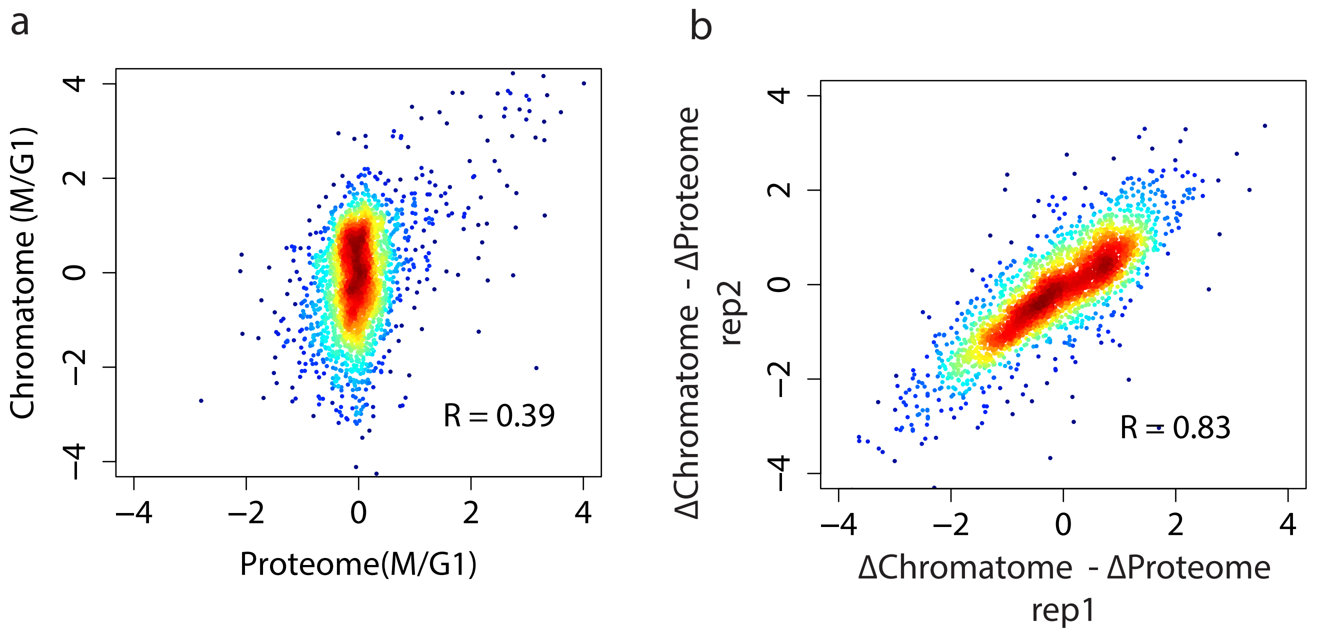

Supplementary Figure 3 Disconnect between chromatin and proteome changes. **a** Contrast of chromatome and proteome changes between mitosis and G1, demonstrating most chromatin changes cannot be explained by a change in protein abundance. **b** Reproducibility of the delta/delta values in panel b. **c** Same as in b, except proteins were binned based on their signal in the proteome. Increasing signal is represented by higher bin number and graphically by the gradient bar below graphs. In each bin, proteins quantified in both the chromatome and proteome were contrasted regarding their changes between G1 and mitosis.

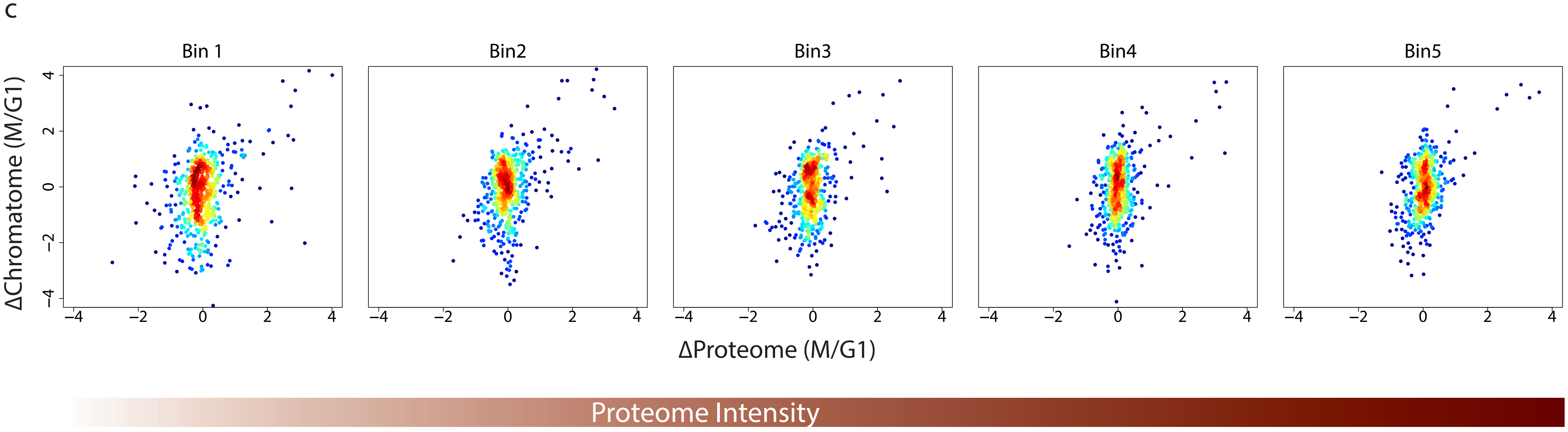

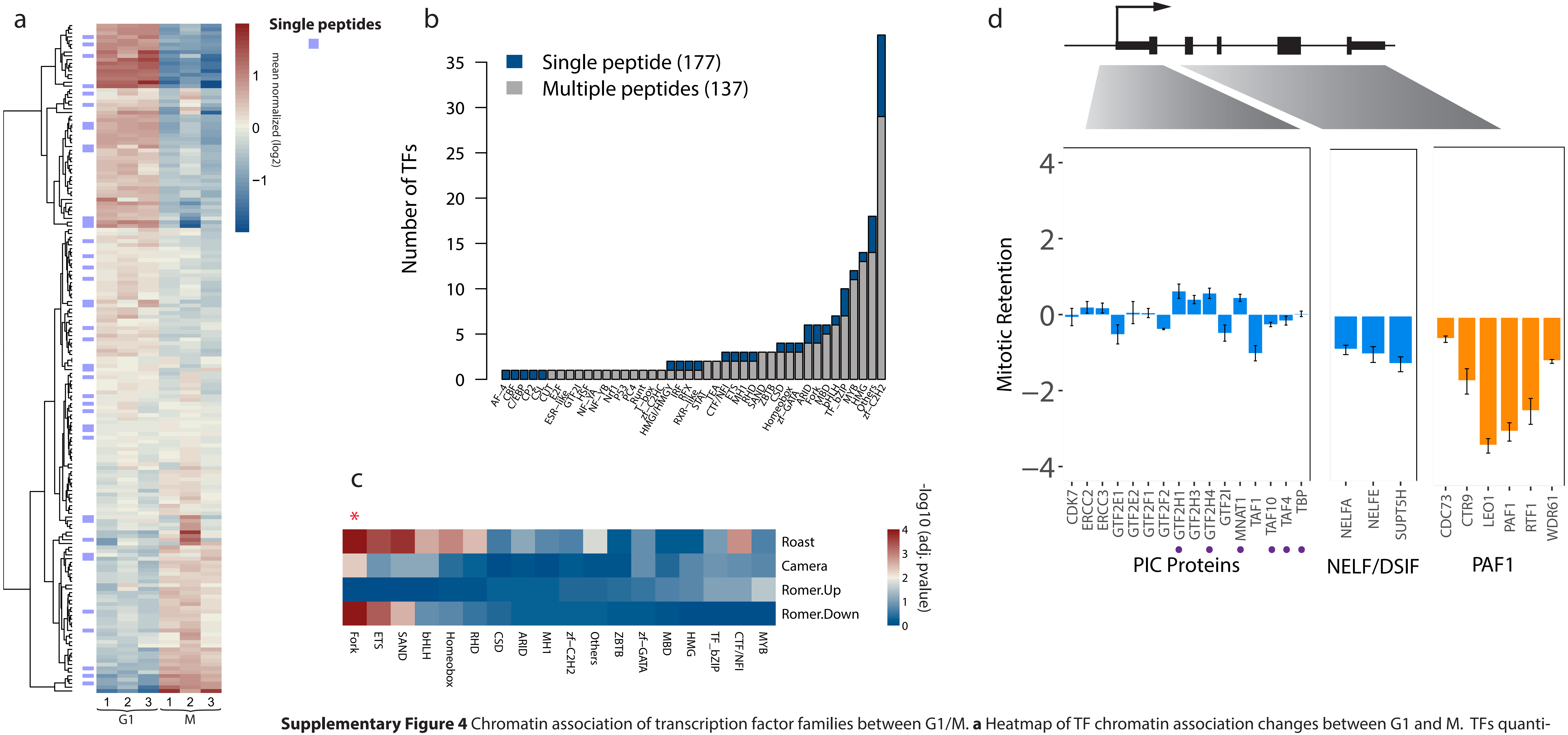

Supplementary Figure 4 Chromatin association of transcription factor families between G1/M. **a** Heatmap of TF chromatin association changes between G1 and M. TFs quantified by a single peptide are marked in the blue annotation column on the left. **b** Frequency of TFs belonging to a particular family. Blue overlay represents additional TFs represented by a single peptide. **c** Heatmap representing -log10(pvals) for enrichment or depletion of TF families from mitotic chromatin (Romer Up and Romer Down respectively, minimum of 3 TFs per family, all quantified TFs). Significance was determined using 3 gene set enrichment strategies from the limma package (mroast87, camera88 and romer89, see methods). Scale is -log10(pval). Forkhead proteins are significant in all metrics (denoted by a red asterisk). **d** Binding behaviour of PIC members, pausing proteins, and the PAF1 complex. Error bars denote standard error of the mean from triplicate measurements, purple dots represent proteins quantified by a single peptide.

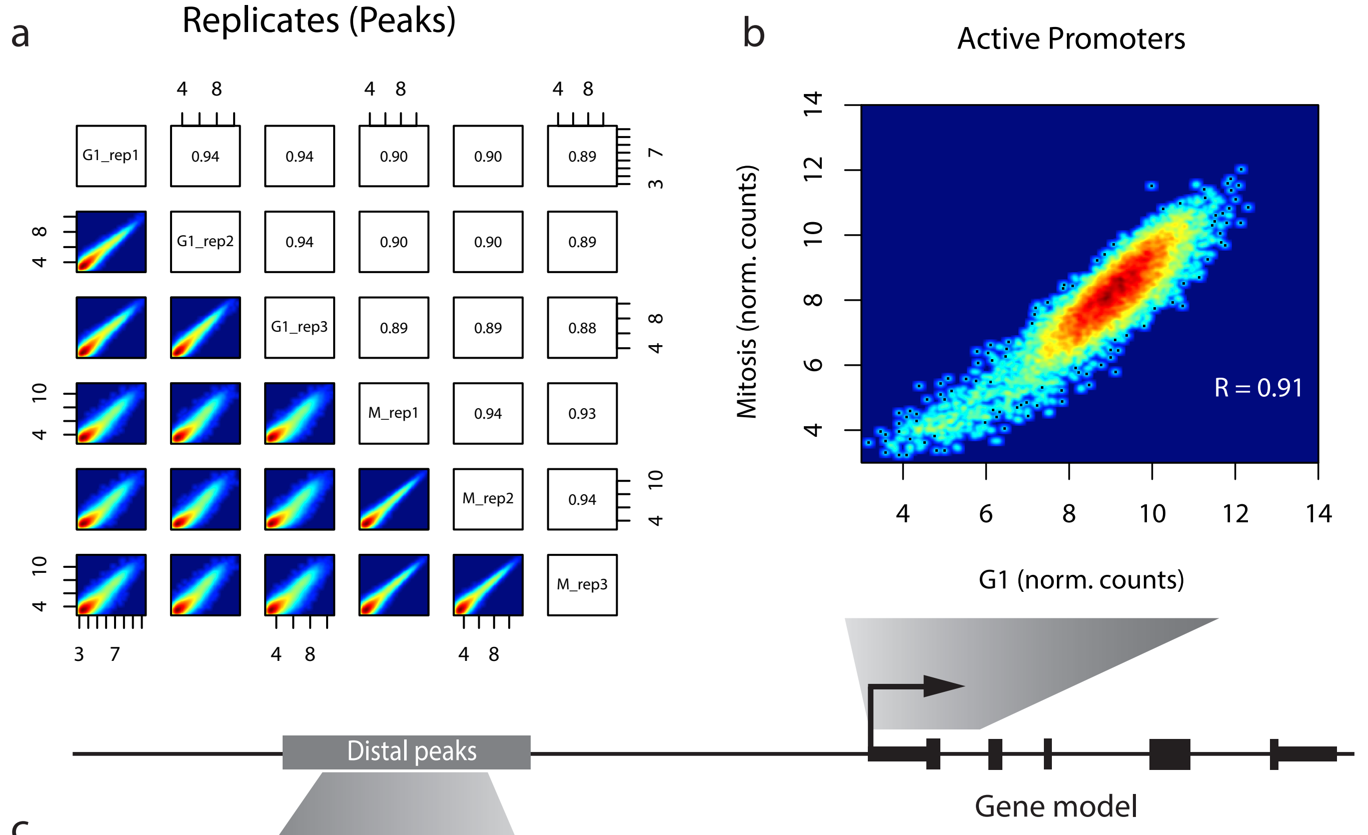

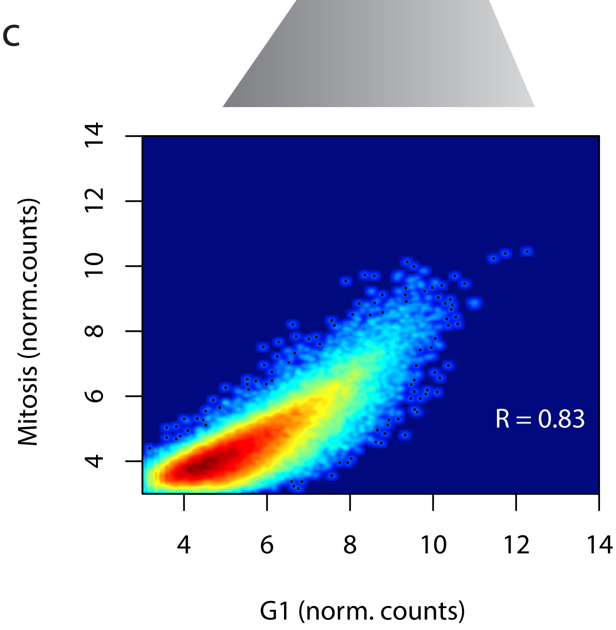

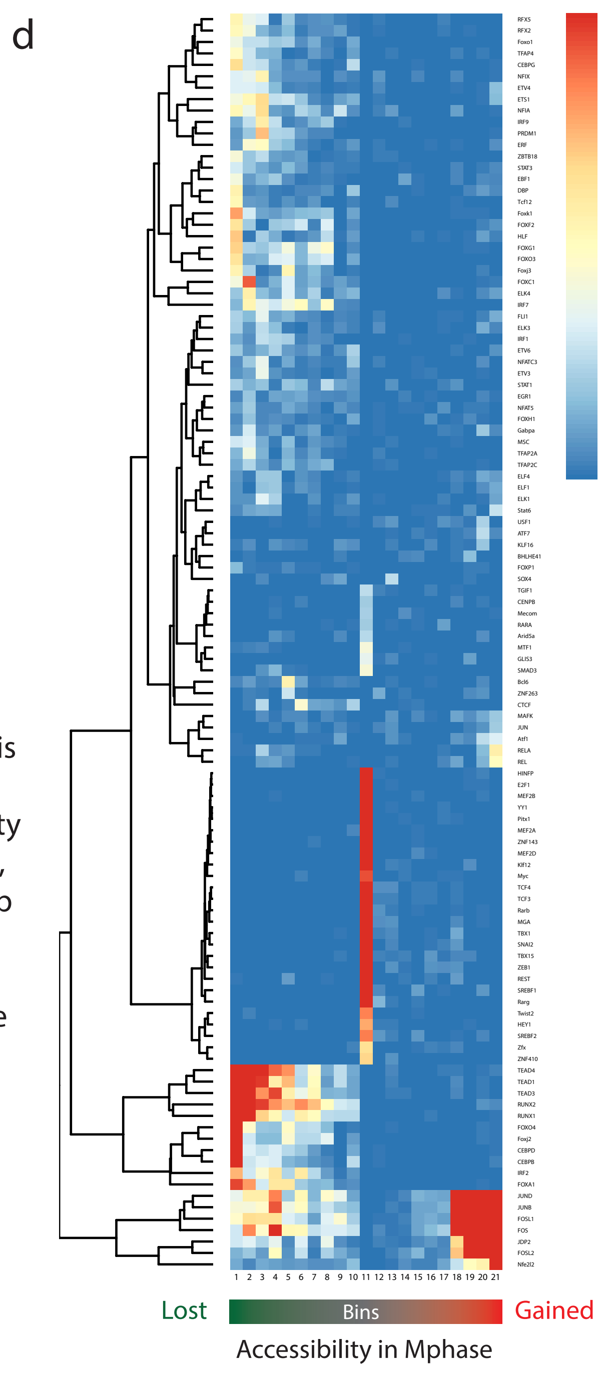

Supplementary Figure 5 The genomic regulatory landscape is largely retained on mitotic chromosomes. **a** Pairs plot of reads in peaks (n = 129,347) for all replicates. Scale is log2(normalized counts). Numbers in the upper boxes represent PCC for the respective comparison. **b** Accessibility at merged promoter peaks (n = 9,414) for G1 and M-phase, scale is as in panel a. **c** Same as in panel b but for sites >2kb from a TSS and no CpG island overlap (n = 61,912). **d** Motif enrichment in distal peaks as a function of accessibility change. Each motif has significant enrichment (adj. pvalue <= 0.05) in at least one bin. TF names for the motifs are noted in each row. Columns represent bins of 500 peaks each, except bin 11 (middle), which contains 51,912 peaks (see panel 5c,d).

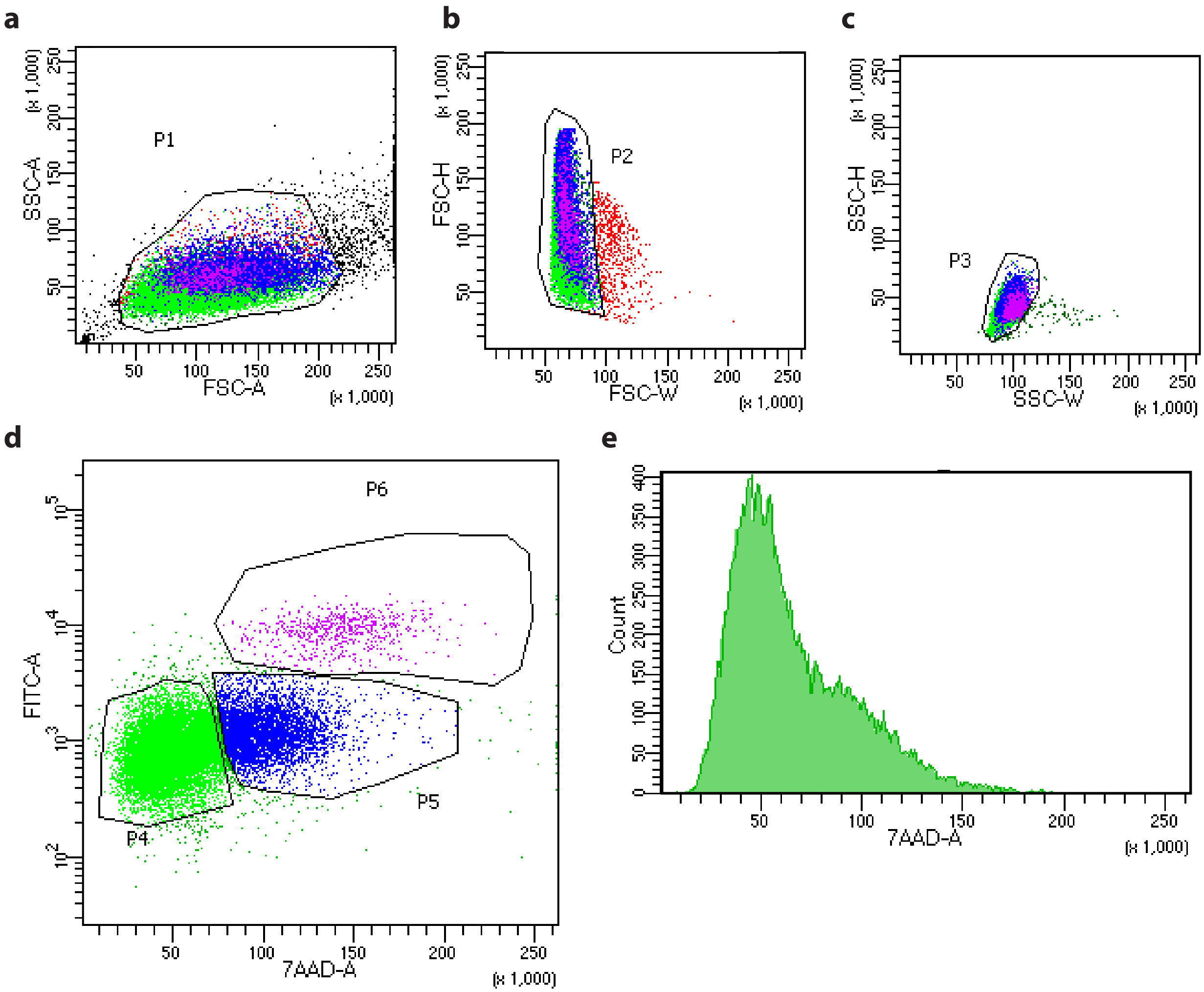

f.

Tube Name: PG20171117_002
SAMPLE ID: Unsync_T11

Population	#Events	%Parent	%Total	DsRed-A Mean	GFP-A Mean
All Events	21,779	####	100.0	85	####
P1	20,793	95.5	95.5	79	####
P2	20,133	96.8	92.4	77	####
P3	20,000	99.3	91.8	77	####
P4	13,708	68.5	62.9	59	####
P5	4,953	24.8	22.7	115	####
P6	636	3.2	2.9	136	####

Supplementary Figure 6 An example of the gating strategy for costaining of unsynchronized cells with 7-AAD and H3T11 phospho antibody. **a** First gate FSC vs SSC was used to exclude debris and dead cells. **b-c** Doublets were excluded with FSC-W vs FSC-H and SSC-W vs SSC-H. **d** Scatter of 7AAD and H3T11 phospho staining (secondary GFP-conjugated antibody). **e** Frequency histogram of 7-AAD staining. **f** Table of cells per gate, points are colored per gate.

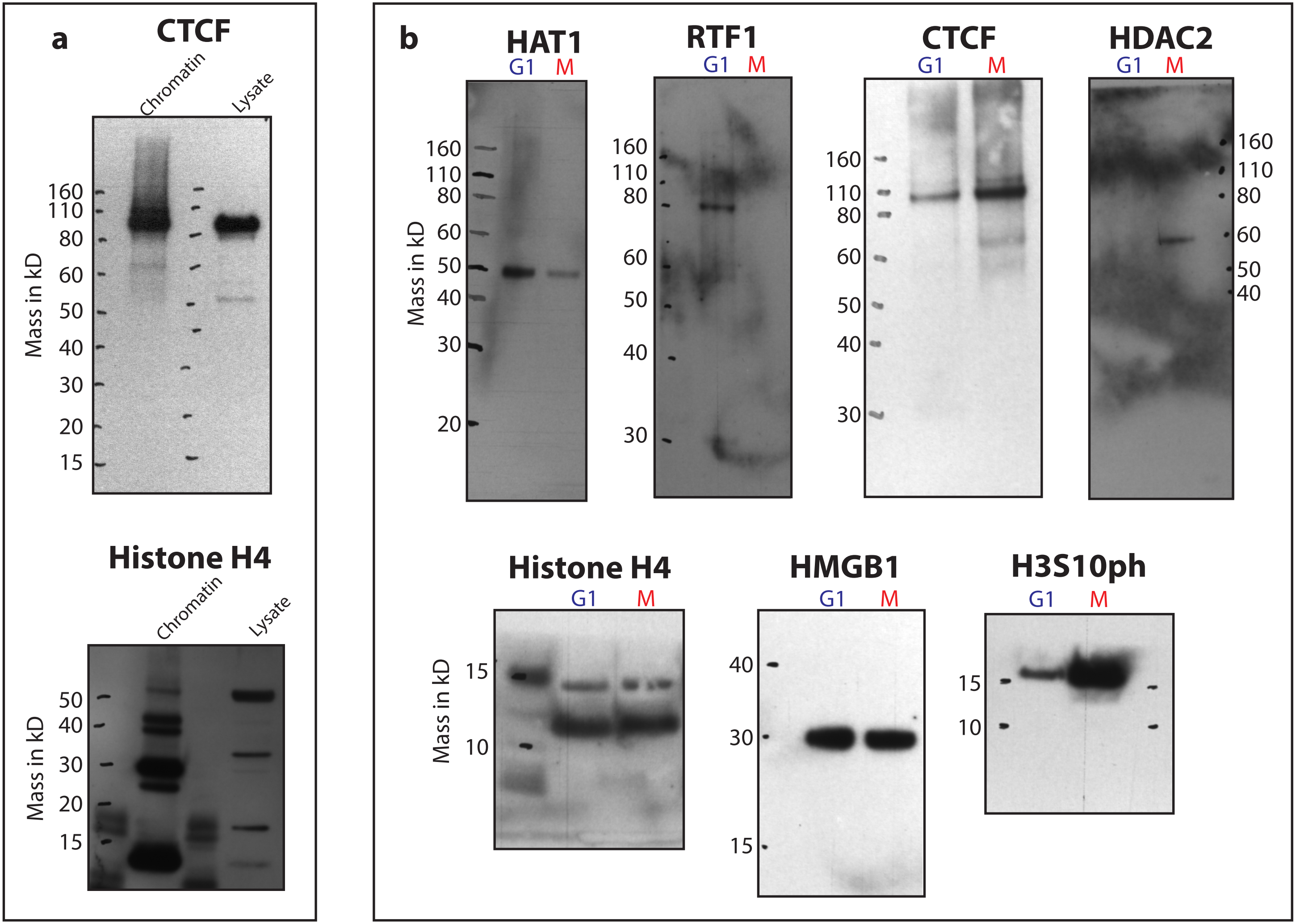

Supplementary Figure 7 Uncropped western blots of proteins from Figure 3d and Supplementary Figure 1b. **a** Western blots of CTCF and Histone H4 from chromatin preps and whole cell lysate (from Supplementary Figure 1b). **b** Western blotfs of proteins from Figure 3d. Approximately 10ug of protein was loaded per lane. Protein names are noted above blots, numbers on the side represent mass in kD based on the protein marker.
