# Supplementary material for: Cell cycle-resolved chromatin proteomics reveals the extent of mitotic preservation of the genomic regulatory landscape
Source: Nat Commun. 2018 Oct 2;9:4048. doi: 10.1038/s41467-018-06007-5 (PMC6168604; doi:10.1038/s41467-018-06007-5)
Supplement: Supplementary file 2 — Description of Additional Supplementary Files [file 41467_2018_6007_MOESM2_ESM.pdf]

Description of Additional Supplementary Files

File Name: Supplementary Data 1
Description: Protein data from subcellular fractionation relevant to Figure 1. Columns with Pr, Nu or Ch represent normalized reporter intensities for Proteome, Nucleome and Chromatome respectively. Other columns include UniProt Accession (Accession), Entrez Gene IDs, Gene Symbols as well as number of total and unique peptides.

File Name: Supplementary Data 2
Description: Cell cycle chromatome and proteome data. Scaled chromatin reporters for triplicate measurements of the synchronized cell cycle stages have a green background, while scaled proteome reporters have a blue background. Uniprot Accession (Accession), Entrez Gene IDs, Gene Symbols as well as peptides per protein are noted.

File Name: Supplementary Data 3
Description: Modified peptide table from the variable modification search in MaxQuant using the cell cycle chromatin data. Replicate measurements for each stage are noted as 'rep'. Reporter intensities represent normalized, log2 converted intensities. The columns Acetyl_K, Trimethyl_K and Phospho_STY represent the number of modification events for each peptide. Score is the Andromeda search engine score, and Delta_score is the Andromeda delta score.
